# Supplementary material for: Systematic profiling of subtelomeric silencing factors in budding yeast
Source: G3 (Bethesda). 2023 Jul 11;13(10):jkad153. doi: 10.1093/g3journal/jkad153 (PMC10542202; doi:10.1093/g3journal/jkad153)
Supplement: jkad153_Supplementary_Data [file jkad153_supplementary_data.zip › Figure_S1_G3-2022-403752.pdf]

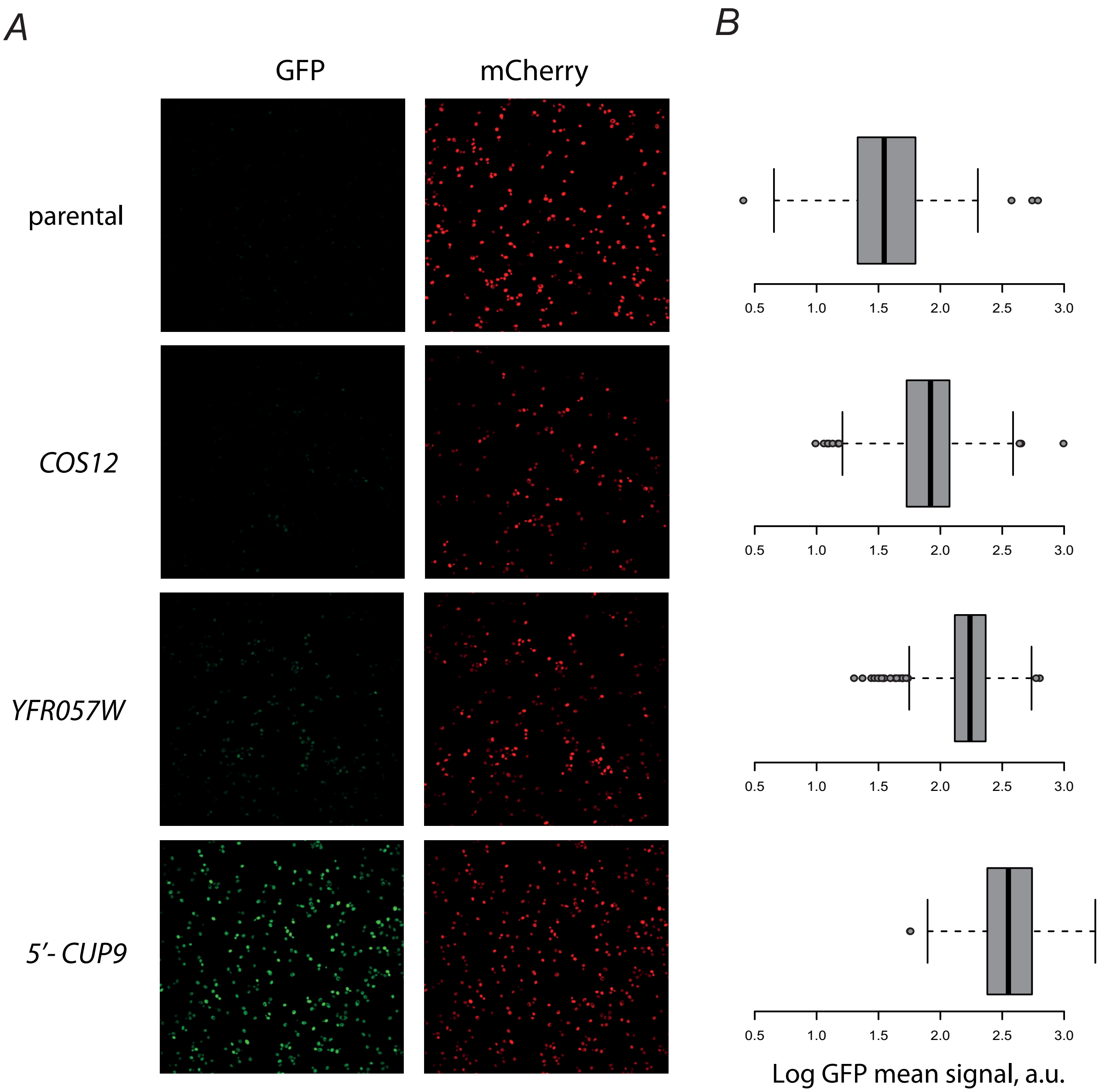

Figure S1. Microscopy analysis of the GFP reporter expressed at different subtelomeric loci. (**A**) Confocal fluorescence microscopy of the Y8205 parental mCherry strain (top) and three different integrations of the *URA3*-GFP reporter at *COS12*, *YFR057W*, and 5'-*CUP9* loci. (**B**) Boxplots show the distributions of median-GFP expression of 1,000 cells captured from combined different fields and analyzed by Cell Profiler. GFP expression is obtained as the mean intensity of pixels inside a gray-scale object, generated for each individual cell after software automatic identification and further processing.
