# Supplementary material for: Systematic profiling of subtelomeric silencing factors in budding yeast
Source: G3 (Bethesda). 2023 Jul 11;13(10):jkad153. doi: 10.1093/g3journal/jkad153 (PMC10542202; doi:10.1093/g3journal/jkad153)
Supplement: jkad153_Supplementary_Data [file jkad153_supplementary_data.zip › Figure_S2_G3-2022-403752.pdf]

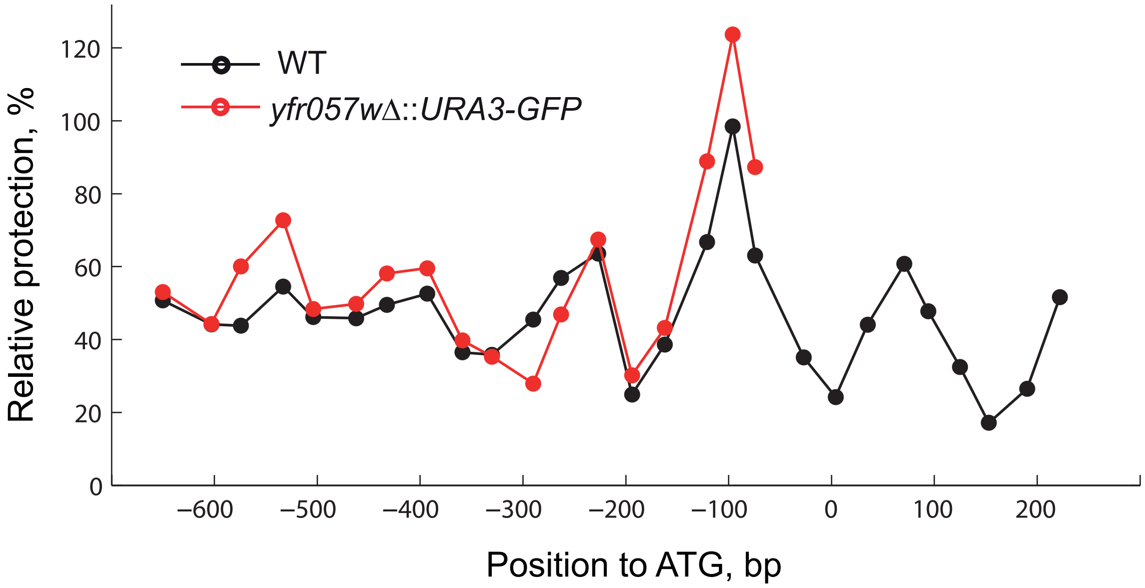

Figure S2. Insertion of the *URA3*-GFP cassette does not alter nucleosome positioning at the *YFR057W* promoter. Nucleosome scanning assay (NuSA) of the *YFR057W* promoter in the parental strain (black line) compared to the strain in which the reporter replaces the *YFR057W* ORF (*yfr057w*Δ::*URA3*-GFP*)* used for subtelomeric screening (red line). Relative protection was calculated relative to the *VCX1* (*YDL128W*) gene where a well-positioned nucleosome is found at the +250 bp position of the ORF. For each of primer pair, midpoint of PCR fragment is shown as a solid dot, and overall amplifying from around -650 to +222 bp of the *YFR057W* locus whose coordinates are given relative to the ATG (+1).
