# Supplementary material for: Systematic profiling of subtelomeric silencing factors in budding yeast
Source: G3 (Bethesda). 2023 Jul 11;13(10):jkad153. doi: 10.1093/g3journal/jkad153 (PMC10542202; doi:10.1093/g3journal/jkad153)
Supplement: jkad153_Supplementary_Data [file jkad153_supplementary_data.zip › Figure_S3_G3-2022-403752.pdf]

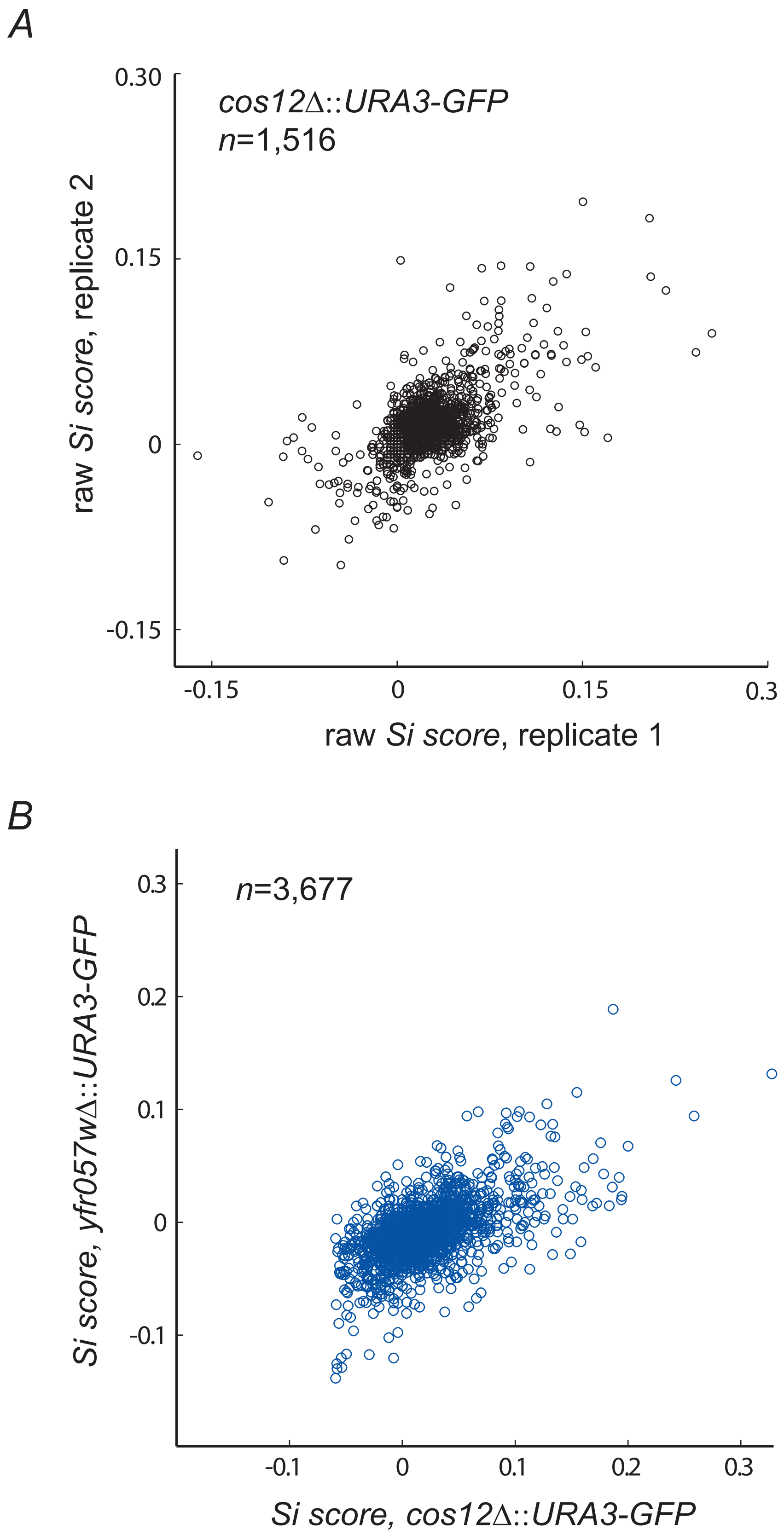

Figure S3. *Si scores* are highly correlated between screens done independently for the same or for different subtelomeric loci. (A) Comparison of *Si scores* obtained from two independent screens performed with a fraction of the mutant collection of subtelomeric *URA3-GFP* at the *COS12 locus* (*n*=1,516, *r*=0.63, $p<10^{-168}$, Spearman). **(B)** Blue dots are the comparison between the *Si scores* obtained from each genomewide silencing screen at the *COS12* and *YFR057W* loci (*n*=3,677; *r*=0.56, $p<10^{-301}$, Spearman).
