# Supplementary material for: Systematic profiling of subtelomeric silencing factors in budding yeast
Source: G3 (Bethesda). 2023 Jul 11;13(10):jkad153. doi: 10.1093/g3journal/jkad153 (PMC10542202; doi:10.1093/g3journal/jkad153)
Supplement: jkad153_Supplementary_Data [file jkad153_supplementary_data.zip › Figure_S4_G3-2022-403752.pdf]

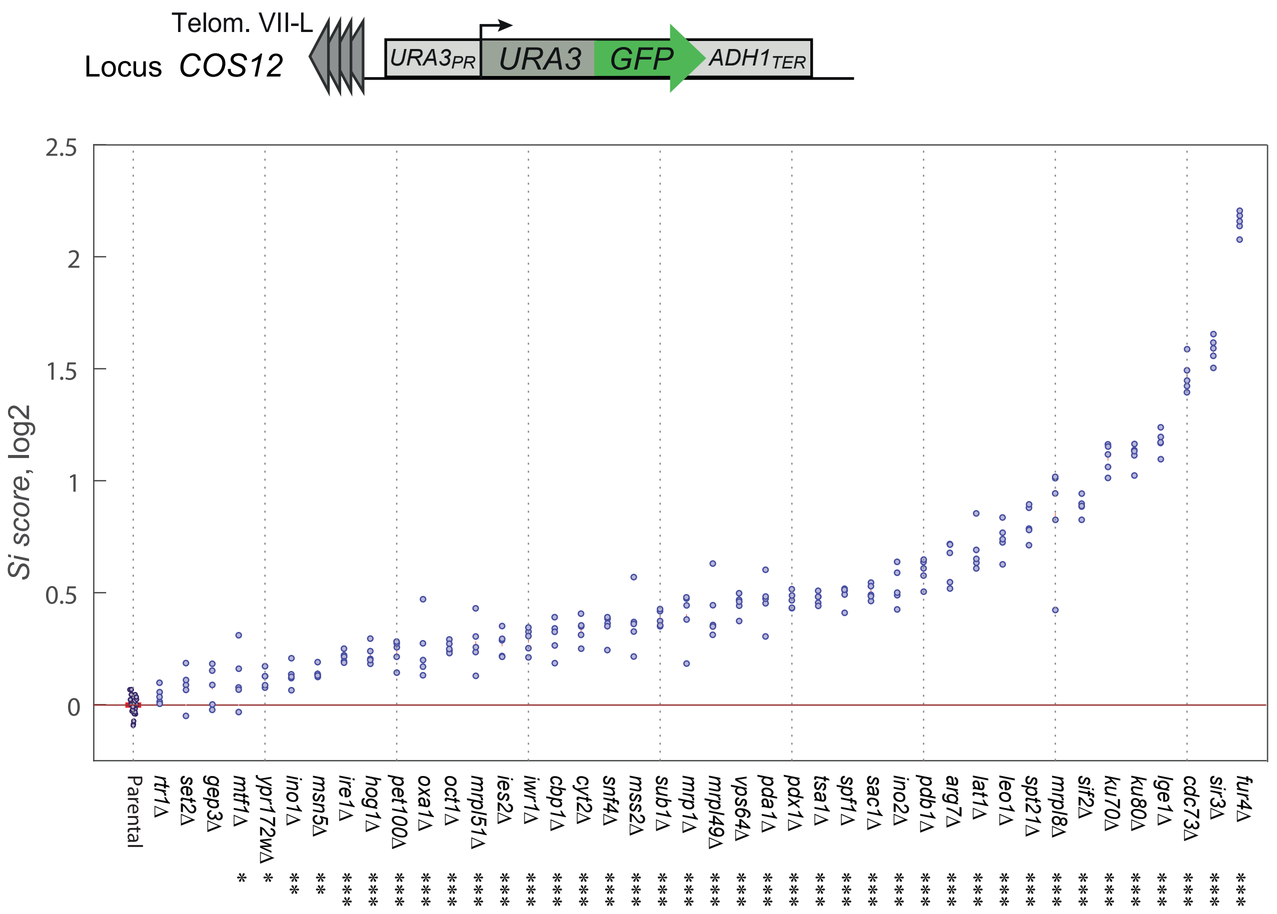

Figure S4. Replicability of *Si scores* obtained for a selected set of mutants. Comparative Si score of representative hits selected from both large-scale silencing screenings with an FDR<10%. The list consists of top-ranked genes by *Si score* (41 genes) and the *sir3*Δ strain as a silencing-factor control. For the *COS12* insertion, five technical replicates were analyzed for each gene-deletion in comparison to the parental strain. Strains were grown on liquid SC medium containing uracil (20 mg/L) for seven hours (log phase) in competition with the corresponding reference strain and then GFP expression was measured by flow cytometry. Each deletion strain was compared to the parental-strain replicates with mean *Si score* equal to zero, by definition (*t*-test; *p<0.05, **p<0.01; ***p<0.005).
