# Supplementary material for: Systematic profiling of subtelomeric silencing factors in budding yeast
Source: G3 (Bethesda). 2023 Jul 11;13(10):jkad153. doi: 10.1093/g3journal/jkad153 (PMC10542202; doi:10.1093/g3journal/jkad153)
Supplement: jkad153_Supplementary_Data [file jkad153_supplementary_data.zip › Figure_S5_G3-2022-403752.pdf]

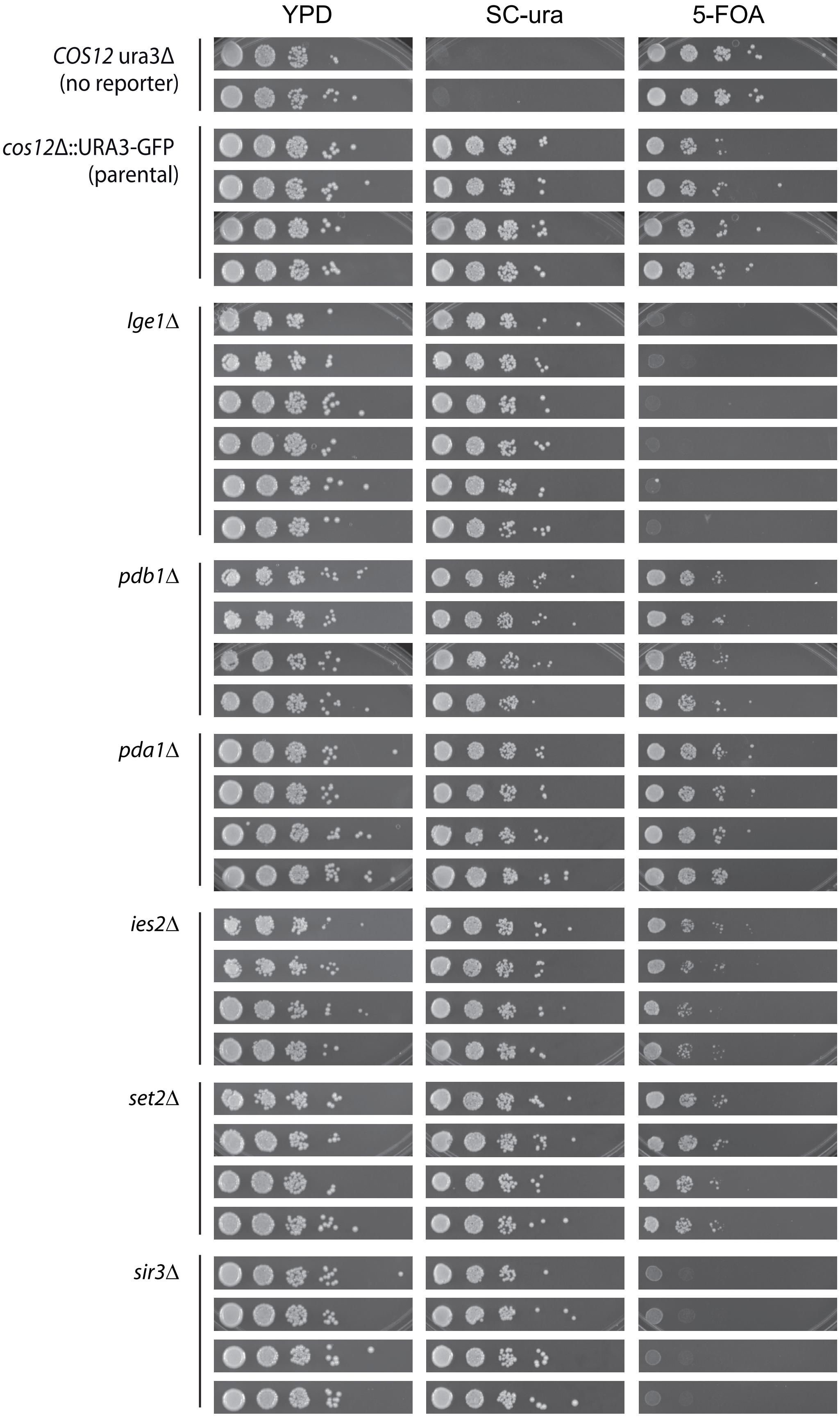

Figure S5. 5-FOA growth assays are highly reproducible. Multiple replicates of histone modification/chromatin remodeling mutants (*lge1*Δ, *sir3*Δ, *set2*Δ, *ies2*Δ), and pyruvate dehydrogenase complex PDC (*pdb1*Δ, *pda1*Δ) were assessed for subtelomeric *URA3* expression on 5-FOA growth assays. Strains were grown overnight on YPD medium and adjusted to OD600nm=1 in water; 10-fold serial dilutions were spotted on YPD, SC-ura and 1.1 g/L 5-FOA agar plates and incubated at 30°C for 48 h. All strains have the URA3-GFP reporter integrated at *COS12* locus, telomere VII-L.
