# Supplementary material for: Systematic profiling of subtelomeric silencing factors in budding yeast
Source: G3 (Bethesda). 2023 Jul 11;13(10):jkad153. doi: 10.1093/g3journal/jkad153 (PMC10542202; doi:10.1093/g3journal/jkad153)
Supplement: jkad153_Supplementary_Data [file jkad153_supplementary_data.zip › Note_S1_G3-2022-403752.pdf]

Supplementary Note S1

A comprehensive list of genes related to subtelomeric silencing was obtained including genes reported in Sacharomyces Genome Database[1] under the synonym terms: (telomeric heterochromatin formation, establishment of chromatin silencing at telomere, telomeric heterochromatin assembly, subtelomeric silencing, chromatin silencing at subtelomere, heterochromatic silencing at subtelomere, subtelomere chromatin silencing, Telomere Position Effect, chromatin silencing at telomere, heterochromatic silencing at telomere, telomere chromatin silencing, telomeric silencing, regulation of chromatin silencing at telomere, regulation of subtelomeric heterochromatin assembly), genes involved in Sir-mediated telomere position effect in *Saccharomyces cerevisiae* listed in Mondoux, M. A. & Zakian, V.A. [2] , and additional genes reported in literature affecting silencing were identified and included.

This list was used as a base for comparison analysis of previously silencing reported genes vs the genes obtained in the current subtelomeric silencing screenings.

Non-essential genes previously related to telomeric silencing in *S. cerevisiae*

Gene name	Systematic name	Description	Ref.
OAF1	*YAL051W*	Oleate-activated transcription factor; acts alone and as a heterodimer with Pip2p; activates genes involved in beta-oxidation of fatty acids and peroxisome organization and biogenesis; OAF1 has a paralog, PIP2, that arose from the whole genome duplication.	3
FUN30	*YAL019W*	Conserved member of the Snf2p family with ATP-dependent chromatin remodeling activity; has a role in silencing; potential Cdc28p substrate; authentic, non-tagged protein is detected in purified mitochondria in high-throughput studies	4
SWD1	*YAR003W*	Subunit of the COMPASS (Set1C) complex, which methylates histone H3 on lysine 4 and is required in transcriptional silencing near telomeres; WD40 beta propeller superfamily member with similarity to mammalian Rbbp7.	5
BRE2	*YLR015W*	Subunit of COMPASS (Set1C) complex, which methylates Lys4 of histone H3 and functions in silencing at telomeres; has a C-terminal Sdc1 Dpy-30 Interaction (SDI) domain that mediates binding to Sdc1p; similar to trithorax-group protein ASH2L	5
SPT21	*YMR179W*	Protein with a role in transcriptional silencing; required for normal transcription at several loci including HTA2-HTB2 and HHF2-HHT2, but not required at the other histone loci; functionally related to Spt10p	6
ESC1	*YMR219W*	Protein localized to the nuclear periphery, involved in telomeric silencing; interacts with PAD4-domain of Sir4p	7

RKR1	*YMR247C*	RING domain E3 ubiquitin ligase; involved in the ubiquitin-mediated degradation of non-stop proteins; functional connections to chromatin modification; nuclear protein that also co-localizes with ribosomes; homolog of mouse Listerin, whose mutation has been reported to cause neurodegeneration in mice	[8]
ZDS1	*YMR273C*	Protein with a role in regulating Swe1p-dependent polarized growth; involved in maintaining Cdc55p in the cytoplasm where it promotes mitotic entry; involved in mitotic exit through Cdc14p regulation; interacts with silencing proteins at the telomere; has a role in Bcy1p localization; implicated in mRNA nuclear export	[9]
YKU70	*YMR284W*	Subunit of the telomeric Ku complex (Yku70p-Yku80p), involved in telomere length maintenance, structure and telomere position effect; relocates to sites of double-strand cleavage to promote nonhomologous end joining during DSB repair	[10]
GAS1	*YMR307W*	Beta-1,3-glucanosyltransferase; required for cell wall assembly and also has a role in transcriptional silencing; localizes to cell surface via a glycosylphosphatidylinositol (GPI) anchor; also found at nuclear periphery; genetic interactions with histone H3 lysine acetyltransferases GCN5 and SAS3 indicate previously unsuspected functions for Gas1 in DNA damage response and cell cycle regulation	[11,12]
RPD3	*YNL330C*	Histone deacetylase; regulates transcription, silencing, and other processes by influencing chromatin remodeling; forms at least two different complexes which have distinct functions and members	[13]
HST3	*YOR025W*	Member of the Sir2 family of NAD(+)-dependent protein deacetylases; involved along with Hst4p in telomeric silencing, cell cycle progression, radiation resistance, genomic stability and short-chain fatty acid metabolism	[14]
HIR2	*YOR038C*	Subunit of the HIR complex, a nucleosome assembly complex involved in regulation of histone gene transcription; recruits Swi-Snf complexes to histone gene promoters; promotes heterochromatic gene silencing with Asf1p	[15]
SNF2	*YOR290C*	Catalytic subunit of the SWI/SNF chromatin remodeling complex involved in transcriptional regulation; contains DNA-stimulated ATPase activity; functions interdependently in transcriptional activation with Snf5p and Snf6p	[16]
ISW2	*YOR304W*	ATP-dependent DNA translocase involved in chromatin remodeling; ATPase component that, with Itc1p, forms a complex required for repression of a-specific genes, INO1, and early meiotic genes during mitotic growth	[17]
SIN3	*YOL004W*	Component of the Sin3p-Rpd3p histone deacetylase complex, involved in transcriptional repression and activation of diverse processes, including mating-type switching and meiosis; involved in the maintenance of chromosomal integrity	[18]

HTZ1	*YOL012C*	Histone variant H2AZ, exchanged for histone H2A in nucleosomes by the SWR1 complex; involved in transcriptional regulation through prevention of the spread of silent heterochromatin	19
ESC8	*YOL017W*	Protein involved in telomeric and mating-type locus silencing, interacts with Sir2p and also interacts with the Gal11p, which is a component of the RNA pol II mediator complex	20
GAL11	*YOL051W*	Subunit of the RNA polymerase II mediator complex; associates with core polymerase subunits to form the RNA polymerase II holoenzyme; affects transcription by acting as target of activators and repressors; forms part of the tail domain of mediator	21
TGS1	*YPL157W*	Trimethyl guanosine synthase, conserved nucleolar methyl transferase that converts the m(7)G cap structure of snRNAs, snoRNAs, and telomerase TLC1 RNA to m(2,2,7)G; also required for nucleolar assembly and splicing of meiotic pre-mRNAs	22
UME1	*YPL139C*	Negative regulator of meiosis, required for repression of a subset of meiotic genes during vegetative growth, binding of histone deacetylase Rpd3p required for activity, contains a NEE box and a WD repeat motif; homologous with Wtm1p, Wtm2p	23
SPP1	*YPL138C*	Subunit of COMPASS (Set1C), a complex which methylates histone H3 on lysine 4 and is required in telomeric transcriptional silencing; interacts with Orc2p; PHD finger domain protein similar to human CGBP, an unmethylated CpG binding protein	5
SWD3	*YBR175W*	Essential subunit of the COMPASS (Set1C) complex, which methylates histone H3 on lysine 4 and is required in transcriptional silencing near telomeres; WD40 beta propeller superfamily member and ortholog of mammalian WDR5	5
PBP2(HEK1)	*YBR233W*	RNA binding protein with similarity to mammalian heterogeneous nuclear RNP K protein, involved in the regulation of telomere position effect and telomere length	24
SHG1	*YBR258C*	Subunit of the COMPASS (Set1C) complex, which methylates histone H3 on lysine 4 and is required in transcriptional silencing near telomeres	5
DPB4	*YDR121W*	Shared subunit of DNA polymerase (II) epsilon and of ISW2/yCHRAC chromatin accessibility complex; involved in both chromosomal DNA replication and in inheritance of telomeric silencing	17
NPL3	*YDR432W*	RNA-binding protein that promotes elongation, regulates termination, and carries poly(A) mRNA from nucleus to cytoplasm; has a role in repressing translation initiation by binding eIF4G; required for pre-mRNA splicing; dissociation from mRNAs promoted by Mtr10p; phosphorylated by Sky1p in the cytoplasm	25
HAT2	*YEL056W*	Subunit of the Hat1p-Hat2p histone acetyltransferase complex; required for high affinity binding of the complex to	26

		free histone H4, thereby enhancing Hat1p activity; similar to human RbAp46 and 48; has a role in telomeric silencing	
ARD1	*YHR013C*	Subunit of protein N-terminal acetyltransferase NatA; NatA is comprised of Nat1p, Ard1p, and Nat5p; acetylates many proteins and thus affects telomeric silencing, cell cycle, heat-shock resistance, mating, and sporulation; human Ard1p levels are elevated in cancer cells; protein abundance increases in response to DNA replication stress (3, 4, 5, 6, 7, 8 and see Summary Paragraph)	[27,28]
SLT2	*YHR030C*	Serine/threonine MAP kinase; involved in regulating maintenance of cell wall integrity, progression through the cell cycle, and nuclear mRNA retention in heat shock; required for mitophagy and pexophagy; affects recruitment of mitochondria to the phagophore assembly site (PAS); regulated by the PKC1-mediated signaling pathway	[29]
RRM3	*YHR031C*	DNA helicase involved in rDNA replication and Ty1 transposition; relieves replication fork pauses at telomeric regions; structurally and functionally related to Pif1p	[30]
UPF2	*YHR077C*	Short telomeres. Protein involved in the nonsense-mediated mRNA decay (NMD) pathway; interacts with Nam7p and Upf3p; involved in telomere maintenance	[31]
MRC1	*YCL061C*	S-phase checkpoint protein required for DNA replication; interacts with and stabilizes Pol2p at stalled replication forks during stress, where it forms a pausing complex with Tof1p and is phosphorylated by Mec1p; protects uncapped telomeres	[32]
PHD1	*YKL043W*	Transcriptional activator that enhances pseudohyphal growth; physically interacts with the Tup1-Cyc8 complex and recruits Tup1p to its targets; regulates expression of FLO11, an adhesin required for pseudohyphal filament formation; similar to StuA, an A. nidulans developmental regulator; potential Cdc28p substrate	[3]
MSN4	*YKL062W*	Transcriptional activator related to Msn2p; activated in stress conditions, which results in translocation from the cytoplasm to the nucleus; binds DNA at stress response elements of responsive genes, inducing gene expression	[3]
ELG1	*YOR144C*	Subunit of an alternative replication factor C complex important for DNA replication and genome integrity; suppresses spontaneous DNA damage; involved in homologous recombination-mediated repair and telomere homeostasis	[33]
NPT1	*YOR209C*	Nicotinate phosphoribosyltransferase, acts in the salvage pathway of NAD+ biosynthesis; required for silencing at rDNA and telomeres and has a role in silencing at mating-type loci; localized to the nucleus	[34]
SAS5	*YOR213C*	Subunit of the SAS complex (Sas2p, Sas4p, Sas5p), which acetylates free histones and nucleosomes and regulates transcriptional silencing; stimulates Sas2p HAT activity	[35]

WTM2	*YOR229W*	Transcriptional modulator involved in regulation of meiosis, silencing, and expression of RNR genes; involved in response to replication stress; contains WD repeats	[23]
WTM1	*YOR230W*	Transcriptional modulator involved in regulation of meiosis, silencing, and expression of RNR genes; required for nuclear localization of the ribonucleotide reductase small subunit Rnr2p and Rnr4p; contains WD repeats	[23]
MKK1	*YOR231W*	Mitogen-activated kinase kinase involved in protein kinase C signaling pathway that controls cell integrity; upon activation by Bck1p phosphorylates downstream target, Slt2p; functionally redundant with Mkk2p	[29]
STE11	*YLR362W*	Signal transducing MEK kinase involved in pheromone response and pseudohyphal/invasive growth pathways where it phosphorylates Ste7p, and the high osmolarity response pathway, via phosphorylation of Pbs2p; regulated by Ste20p and Ste50p	[36]
MEC3	*YLR288C*	DNA damage and meiotic pachytene checkpoint protein; subunit of a heterotrimeric complex (Rad17p-Mec3p-Ddc1p) that forms a sliding clamp, loaded onto partial duplex DNA by a clamp loader complex; homolog of human and S. pombe Hus1	[37]
SAS4	*YDR181C*	Subunit of the SAS complex (Sas2p, Sas4p, Sas5p), which acetylates free histones and nucleosomes and regulates transcriptional silencing; required for the HAT activity of Sas2p	[35]
HST4	*YDR191W*	Member of the Sir2 family of NAD(+)-dependent protein deacetylases; involved along with Hst3p in silencing at telomeres, cell cycle progression, radiation resistance, genomic stability and short-chain fatty acid metabolism	[14]
ADR1	*YDR216W*	Carbon source-responsive zinc-finger transcription factor, required for transcription of the glucose-repressed gene ADH2, of peroxisomal protein genes, and of genes required for ethanol, glycerol, and fatty acid utilization	[3]
HTA1	*YDR225W*	Histone H2A	[38]
SIR4	*YDR227W*	Silent information regulator that, together with SIR2 and SIR3, is involved in assembly of silent chromatin domains at telomeres and the silent mating-type loci; potentially phosphorylated by Cdc28p; some alleles of SIR4 prolong lifespan	[28]
RTF1	*YGL244W*	Subunit of RNAPII-associated chromatin remodeling Paf1 complex; regulates gene expression by directing cotranscriptional histone modification, influences transcription and chromatin structure through several independent functional domains; directly or indirectly regulates DNA-binding properties of Spt15p and relative activities of different TATA elements; involved in transcription elongation as demonstrated by the G-less-based run-on (GLRO) assay	[5,39]
ELP3	*YPL086C*	Subunit of Elongator complex, which is required for modification of wobble nucleosides in tRNA; exhibits histone	[40]

		acetyltransferase activity that is directed to histones H3 and H4; disruption confers resistance to K. lactis zymotoxin	
HST2	*YPL015C*	Cytoplasmic member of the silencing information regulator 2 (Sir2) family of NAD(+)-dependent protein deacetylases; modulates nucleolar (rDNA) and telomeric silencing; possesses NAD(+)-dependent histone deacetylase activity in vitro	41
HAT1	*YPL001W*	Catalytic subunit of the Hat1p-Hat2p histone acetyltransferase complex that uses the cofactor acetyl coenzyme A, to acetylate free nuclear and cytoplasmic histone H4; involved in telomeric silencing and DNA double-strand break repair	26
HTA2	*YBL003C*	Histone H2A, core histone protein required for chromatin assembly and chromosome function; one of two nearly identical (see also HTA1) subtypes; DNA damage-dependent phosphorylation by Mec1p facilitates DNA repair; acetylated by Nat4p	38
HIR1	*YBL008W*	Subunit of the HIR complex, a nucleosome assembly complex involved in regulation of histone gene transcription; contributes to nucleosome formation, heterochromatic gene silencing, and formation of functional kinetochores	15
FUS3	*YBL016W*	Mitogen-activated serine/threonine protein kinase involved in mating; phosphoactivated by Ste7p; substrates include Ste12p, Far1p, Bni1p, Sst2p; inhibits invasive growth during mating by phosphorylating Tec1p, promoting its degradation	36
SAS3	*YBL052C*	Histone acetyltransferase catalytic subunit of NuA3 complex that acetylates histone H3, involved in transcriptional silencing; homolog of the mammalian MOZ proto-oncogene; mutant has aneuploidy tolerance; sas3gcn5 double mutation is lethal	42
PNC1	*YGL037C*	Nicotinamidase that converts nicotinamide to nicotinic acid as part of the NAD(+) salvage pathway, required for life span extension by calorie restriction; PNC1 expression responds to all known stimuli that extend replicative life span	34
RAD6	*YGL058W*	Ubiquitin-conjugating enzyme (E2), involved in postreplication repair (as a heterodimer with Rad18p), DSBR and checkpoint control (as a heterodimer with Bre1p), ubiquitin-mediated N-end rule protein degradation (as a heterodimer with Ubr1p	43
RTT106	*YNL206C*	Histone chaperone, involved in regulation of chromatin structure in both transcribed and silenced chromosomal regions; affects transcriptional elongation; has a role in regulation of Ty1 transposition	44
SUM1	*YDR310C*	Transcriptional repressor required for mitotic repression of middle sporulation-specific genes; also acts as general replication initiation factor; involved in telomere maintenance, chromatin silencing; regulated by pachytene checkpoint	45
DOT5	*YIL010W*	Nuclear thiol peroxidase which functions as an alkyl-hydroperoxide reductase during post-diauxic growth	46
YAP5	*YIR018W*	Basic leucine zipper (bZIP) iron-sensing transcription factor	3

DOT6	*YER088C*	Protein involved in rRNA and ribosome biogenesis; binds polymerase A and C motif; subunit of the RPD3L histone deacetylase complex; similar to Tod6p; has chromatin specific SANT domain; involved in telomeric gene silencing and filamentation	46
IES3	*YLR052W*	Subunit of the INO80 chromatin remodeling complex	47
CAC2	*YML102W*	Subunit of chromatin assembly factor I (CAF-1), with Rlf2p and Msi1p; chromatin assembly by CAF-1 is important for multiple processes including silencing at telomeres, mating type loci, and rDNA; maintenance of kinetochore structure; deactivation of the DNA damage checkpoint after DNA repair; and chromatin dynamics during transcription	48
ZDS2	*YML109W*	Protein with a role in regulating Swe1p-dependent polarized growth; involved in maintenance of Cdc55p in the cytoplasm where it promotes mitotic entry; interacts with silencing proteins at the telomere; implicated in the mitotic exit network through regulation of Cdc14p localization; paralog of Zds1p	9
GTR1	*YML121W*	Cytoplasmic GTP binding protein and negative regulator of the Ran/Tc4 GTPase cycle; component of GSE complex, which is required for sorting of Gap1p; involved in phosphate transport and telomeric silencing; similar to human RagA and RagB	49
YKU80	*YMR106C*	Subunit of the telomeric Ku complex (Yku70p-Yku80p), involved in telomere length maintenance, structure and telomere position effect; relocates to sites of double-strand cleavage to promote nonhomologous end joining during DSB repair	10
SAS2	*YMR127C*	Histone acetyltransferase (HAT) catalytic subunit of the SAS complex (Sas2p-Sas4p-Sas5p), which acetylates free histones and nucleosomes and regulates transcriptional silencing; member of the MYSTacetyltransferase family	50,51
ROX1	*YPR065W*	Heme-dependent repressor of hypoxic genes; contains an HMG domain that is responsible for DNA bending activity	3
SPT10	*YJL127C*	Putative histone acetylase with a role in transcriptional silencing	52
ASF1	*YJL115W*	Nucleosome assembly factor, involved in chromatin assembly and disassembly, anti-silencing protein that causes derepression of silent loci when overexpressed; plays a role in regulating Ty1 transposition	53
BCK1	*YJL095W*	Mitogen-activated protein (MAP) kinase kinase kinase acting in the protein kinase C signaling pathway, which controls cell integrity; upon activation by Pkc1p phosphorylates downstream kinases Mkk1p and Mkk2p	29
TDH3	*YGR192C*	Glyceraldehyde-3-phosphate dehydrogenase (GAPDH), isozyme 3; involved in glycolysis and gluconeogenesis; tetramer that catalyzes the reaction of glyceraldehyde-3-phosphate to 1,3 bis-phosphoglycerate; detected in the cytoplasm and cell wall; GAPDH-derived antimicrobial	54

		peptides secreted by S. cerevisiae are active against a wide variety of wine-related yeasts and bacteria; binds AU-rich RNA	
DLS1	*YJL065C*	Subunit of ISW2/yCHRAC chromatin accessibility complex along with Itc1p, Isw2p, and Dpb4p; involved in inheritance of telomeric silencing	[17]
SIR1	*YKR101W*	Protein involved in repression of transcription at the silent mating-type loci HML and HMR; recruitment to silent chromatin requires interactions with Orc1p and with Sir4p, through a common Sir1p domain; binds to centromeric chromatin	[55]
RIF2	*YLR453C*	Protein that binds to the Rap1p C-terminus and acts synergistically with Rif1p to help control telomere length and establish telomeric silencing; deletion results in telomere elongation	[56]
MOG1	*YJR074W*	Conserved nuclear protein that interacts with GTP-Gsp1p	[57]
DPB3	*YBR278W*	Third-largest subunit of DNA polymerase II (DNA polymerase epsilon), required to maintain fidelity of chromosomal replication and also for inheritance of telomeric silencing; mRNA abundance peaks at the G1/S boundary of the cell cycle	[17]
POL32	*YJR043C*	Third subunit of DNA polymerase delta, involved in chromosomal DNA replication; required for error-prone DNA synthesis in the presence of DNA damage and processivity; interacts with Hys2p, PCNA (Pol30p), and Pol1p	[58]
STE7	*YDL159W*	Signal transducing MAP kinase kinase involved in pheromone response, where it phosphorylates Fus3p, and in the pseudohyphal/invasive growth pathway, through phosphorylation of Kss1p; phosphorylated by Ste11p, degraded by ubiquitin pathway	[36]
HHF2	*YNL030W*	Histone H4, core histone protein required for chromatin assembly and chromosome function; one of two identical histone proteins (see also HHF1); contributes to telomeric silencing; N-terminal domain involved in maintaining genomic integrity	[28,59,60]
HHT2	*YNL031C*	Histone H3, core histone protein required for chromatin assembly, part of heterochromatin-mediated telomeric and HM silencing; one of two identical histone H3 proteins (see HHT1); regulated by acetylation, methylation, and phosphorylation	[59,60]
HHF1	*YBR009C*	Histone H4, core histone protein required for chromatin assembly and chromosome function; one of two identical histone proteins (see also HHF2); contributes to telomeric silencing; N-terminal domain involved in maintaining genomic integrity	[28,59,60]
HHT1	*YBR010W*	Histone H3, core histone protein required for chromatin assembly, part of heterochromatin-mediated telomeric and HM silencing; one of two identical histone H3 proteins (see HHT2); regulated by acetylation, methylation, and phosphorylation	[59,60]

YAF9	*YNL107W*	Subunit of both the NuA4 histone H4 acetyltransferase complex and the SWR1 complex, may function to antagonize silencing near telomeres; interacts directly with Swc4p, has homology to human leukemogenic protein AF9, contains a YEATS domain	[61]
SPT4	*YGR063C*	Component of the universally conserved Spt4/5 complex (DSIF complex); the complex has multiple roles in concert with RNA polymerases I and II, including regulation of transcription elongation, RNA processing, quality control, and transcription-coupled DNA repair; Spt4p also localizes to kinetochores and heterochromatin and affects chromosome dynamics and silencing; required for transcription through lengthy trinucleotide repeats in ORFs or non-protein coding regions	[62]
RIF1	*YBR275C*	Protein that binds to the Rap1p C-terminus and acts synergistically with Rif2p to help control telomere length and establish telomeric silencing; deletion results in telomere elongation	[56]
SIR3	*YLR442C*	Silencing protein that interacts with Sir2p and Sir4p, and histone H3 and H4 tails, to establish a transcriptionally silent chromatin state; required for spreading of silenced chromatin; recruited to chromatin through interaction with Rap1p	[28]
SLX5	*YDL013W*	Subunit of the Slx5-Slx8 SUMO-targeted ubiquitin ligase (STUbL) complex, stimulated by SUMO-modified substrates; contains a RING domain and two SIMs (SUMO-interacting motifs); forms SUMO-dependent nuclear foci, including DNA repair centers	[63]
SIR2	*YDL042C*	Conserved NAD+ dependent histone deacetylase of the Sirtuin family; involved in regulation of lifespan; plays roles in silencing at HML, HMR, telomeres, and the rDNA locus; negatively regulates initiation of DNA replication; functions as a regulator of autophagy like mammalian homolog SIRT1, and also of mitophagy	[28]
DOT1	*YDR440W*	Nucleosomal histone H3-Lys79 methylase; methylation is required for telomeric silencing, meiotic checkpoint control, and DNA damage response	[46]
ADA2	*YDR448W*	Transcription coactivator, component of the ADA and SAGA transcriptional adaptor/HAT (histone acetyltransferase) complexes	[64]
SDC1	*YDR469W*	Subunit of the COMPASS (Set1C) complex, which methylates lysine 4 of histone H3 and is required in chromatin silencing at telomeres; contains a Dpy-30 domain that mediates interaction with Bre2p; similar to C. elegans and human DPY-30	[5]
ITC1	*YGL133W*	Subunit of the ATP-dependent Isw2p-Itc1p chromatin remodeling complex, required for repression of a-specific genes, repression of early meiotic genes during mitotic growth, and repression of INO1; similar to mammalian Acf1p, the regulatory subunit of the mammalian ATP-	[17]

		utilizing chromatin assembly and modifying factor (ACF) complex	
SCS2	*YER120W*	Integral ER membrane protein that regulates phospholipid metabolism via an interaction with the FFAT motif of Opi1p, also involved in telomeric silencing, disruption causes inositol auxotrophy above 34 degrees C, VAP homolog	[65]
BRE1	*YDL074C*	E3 ubiquitin ligase, forms heterodimer with Rad6p to monoubiquinate histone H2B-K123, which is required for the subsequent methylation of histone H3-K4 and H3-K79; required for DSBR, transcription, silencing, and checkpoint control	[66]
BDF1	*YLR399C*	Protein involved in transcription initiation; functions at TATA-containing promoters; associates with the basal transcription factor TFIID; contains two bromodomains; corresponds to the C-terminal region of mammalian TAF1; redundant with Bdf2p.	[67]
NAT1	*YDL040C*	Subunit of protein N-terminal acetyltransferase NatA; NatA is comprised of Nat1p, Ard1p, and Nat5p; N-terminally acetylates many proteins, which influences multiple processes such as the cell cycle, heat-shock resistance, mating, sporulation, and telomeric silencing.	[28]
SAP30	*YMR263W*	Subunit of a histone deacetylase complex, along with Rpd3p and Sin3p, that is involved in silencing at telomeres, rDNA, and silent mating-type loci; involved in telomere maintenance.	[18]
DIA2	*YOR080W*	Origin-binding F-box protein; forms SCF ubiquitin ligase complex with Skp1p and Cdc53p; functions in ubiquitination of silent chromatin structural protein Sir4p; required to target Cdc6p for destruction during G1 phase; required for deactivation of Rad53 checkpoint kinase, completion of DNA replication during recovery from DNA damage, assembly of RSC complex, RSC-mediated transcription regulation, and nucleosome positioning; involved in invasive and pseudohyphal growth.	[68]
IFH1	*YLR223C*	Coactivator, regulates transcription of ribosomal protein (RP) genes; recruited to RP gene promoters during optimal growth conditions via Fhl1p; subunit of CURI, a complex that coordinates RP production and pre-rRNA processing; regulated by acetylation and phosphorylation at different growth states via TORC1 signaling; IFH1 has a paralog, CRF1, that arose from the whole genome duplication.	[46]
INO80	*YGL150C*	Nucleosome spacing factor; subunit of complex containing actin and actin-related proteins that has chromatin remodeling activity and 3' to 5' DNA helicase activity; involved in positioning and spacing of nucleosomes over most genes independently of transcription; promotes nucleosome shifts in the 3' direction; has a role in modulating stress gene transcription	[49]
MCM10	*YIL150C*	Essential chromatin-associated protein; involved in initiation of DNA replication; required for association of MCM2-7	[69]

		complex with replication origins; required to stabilize catalytic subunit of DNA polymerase-alpha; coordinates function of replication fork helicase; self-associates through its N-terminal domain	
MCM3	*YEL032W*	Protein involved in DNA replication; component of the Mcm2-7 hexameric helicase complex that binds chromatin as a part of the pre-replicative complex	[70]
MCM7	*YBR202W*	Component of the Mcm2-7 hexameric helicase complex; MCM2-7 primes origins of DNA replication in G1 and becomes an active ATP-dependent helicase that promotes DNA melting and elongation in S-phase; forms an Mcm4p-6p-7p subcomplex	[70]
MPS3	*YJL019W*	Nuclear envelope protein; required for SPB insertion, SPB duplication, Kar5p localization near the SPB and nuclear fusion; interacts with Mps2p to tether half-bridge to core SPB; N-terminal acetylation by Eco1p regulates its role in nuclear organization; localizes to the SPB half bridge and telomeres during meiosis; required with Ndj1p and Csm4p for meiotic bouquet formation and telomere-led rapid prophase movement; member of the SUN protein family (Sad1-UNC-84 homology).	[71]
RNA1	*YMR235C*	GTPase activating protein (GAP) for Gsp1p; involved in nuclear transport.	[72]
SCP160	*YJL080C*	Essential RNA-binding G protein effector of mating response pathway; ligand-activated RNA-binding protein that delivers RNAs involved in polarization and perpetualizing mating signal to shmoo tip during pheromone signaling; Scp160p-mediated RNA trafficking essential for chemotropism and successful mating; mainly associated with nuclear envelope and ER, interacts in mRNA-dependent manner with translating ribosomes via multiple KH domains, similar to vertebrate vigilins	[73]
SUB2	*YDL084W*	Component of the TREX complex required for nuclear mRNA export; member of the DEAD-box RNA helicase superfamily and is involved in early and late steps of spliceosome assembly; homolog of the human splicing factor hUAP56; relocalizes from nucleus to cytoplasm upon DNA replication stress.	[74]
RLF2 (CAC1)	*YPR018W*	Largest subunit (p90) of the Chromatin Assembly Complex (CAF-1); chromatin assembly by CAF-1 is important for multiple processes including histone H3/H4 tetramerization, silencing at telomeres, mating type loci and rDNA, maintenance of kinetochore structure, deactivation of DNA damage checkpoint after DNA repair, chromatin dynamics during transcription, and repression of divergent noncoding transcription.	[75,76]
MSI1 (CAC3)	*YBR195C*	Subunit of chromatin assembly factor I (CAF-1); chromatin assembly by CAF-1 affects multiple processes including silencing at telomeres, mating type loci, and rDNA; maintenance of kinetochore structure; deactivation of DNA damage checkpoint after DNA repair; chromatin dynamics	[76]

		during transcription; and repression of divergent noncoding transcription; Msi1p localizes to nucleus and cytoplasm and independently regulates the RAS/cAMP pathway via sequestration of Npr1p kinase.	
GZF3	*YJL110C*	GATA zinc finger protein; negatively regulates nitrogen catabolic gene expression by competing with Gat1p for GATA site binding; function requires a repressive carbon source; dimerizes with Dal80p and binds to Tor1p; GZF3 has a paralog, DAL80, that arose from the whole genome duplication.	[3]

Essential genes previously related to telomeric silencing in *S. cerevisiae*

Gene name	Systematic name	Description	Ref.
HIF1	*YLL022C*	Non-essential component of the HAT-B histone acetyltransferase complex; localized to the nucleus; has a role in telomeric silencing; other members are Hat1p and Hat2p.	[77,78]
MCM5	*YLR274W*	Component of the Mcm2-7 hexameric helicase complex; MCM complex is important for priming origins of DNA replication in G1 and becomes an active ATP-dependent helicase that promotes DNA melting and elongation when activated by Cdc7p-Dbf4p in S-phase.	[79]
ORC2	*YBR060C*	Subunit of the origin recognition complex (ORC); ORC directs DNA replication by binding to replication origins and is also involved in transcriptional silencing; interacts with Spp1p and with trimethylated histone H3; phosphorylated by Cdc28p.	[79]
POL30 (PCNA)	*YBR088C*	Proliferating cell nuclear antigen (PCNA); functions as the sliding replication clamp for DNA polymerase delta; may function as a docking site for other proteins required for mitotic and meiotic chromosomal DNA replication and for DNA repair; PCNA ubiquitination at K164 plays a crucial role during Okazaki fragment processing.	[80]
RAP1	*YNL216W*	Essential DNA-binding transcription regulator that binds many loci; involved in transcription activation, repression, chromatin silencing, telomere length maintenance; represses divergent noncoding transcription at highly expressed genes, prevents transcription initiation at cryptic promoters near its binding sites; relocalizes to cytosol under hypoxia; recruits Sir complex to telomeric DNA; present in quiescent cell telomere hyperclusters.	[81,82]
SET1	*YHR119W*	Histone methyltransferase, subunit of the COMPASS (Set1C) complex; COMPASS methylates histone H3K4; Set1p-dependent H3K4 trimethylation recruits Nrd1p, allowing efficient termination of snoRNAs and cryptic unstable	[37,83-85]

		transcripts (CUTs) by Nrd1p-Nab3p-Sen1p pathway; modulates histone acetylation levels in promoter proximal regions to ensure efficient Nrd1p-dependent termination; required in transcriptional silencing near telomeres and at silent mating type loci; has a SET domain.	
UBP10	*YNL186W*	Ubiquitin-specific protease, deubiquitinates Ub-protein moieties; interacts with proteins that function in rRNA production and ribosome biogenesis via its intrinsically disordered regions; stabilizes Rpa190p by deubiquitination; controls PCNA deubiquitylation; may regulate silencing by acting on Sir4p; involved in posttranscriptionally regulating Gap1p, possibly other transporters; localized to the nucleolus; null mutant phenotypes are functionally complemented by human USP36.	[86,87]
ORC5	*YNL261W*	Subunit of the origin recognition complex (ORC); ORC directs DNA replication by binding to replication origins and is also involved in transcriptional silencing.	[88]
ABF1	*YKL112W*	DNA binding protein with possible chromatin-reorganizing activity; involved in transcriptional activation, gene silencing, and DNA replication and repair.	[55]
SWD2	*YKL018W*	Subunit of the COMPASS (Set1C) histone H3K4 methyltransferase complex; required for Set1C stability and optimal activity; COMPASS methylates histone H3 on lys 4 and is involved in telomeric silencing; subunit of CPF (cleavage and polyadenylation factor), a complex involved in RNAP II transcription termination.	[84]
PKC1	*YBL105C*	Protein serine/threonine kinase; essential for cell wall remodeling during growth; localized to sites of polarized growth and the mother-daughter bud neck; homolog of the alpha, beta, and gamma isoforms of mammalian protein kinase C (PKC).	[29]
STE12	*YHR084W*	Transcription factor that is activated by a MAPK signaling cascade; activates genes involved in mating or pseudohyphal/invasive growth pathways; cooperates with Tec1p transcription factor to regulate genes specific for invasive growth.	[36]
EPL1	*YFL024C*	Subunit of NuA4, an essential histone H4/H2A acetyltransferase complex; conserved region at N-terminus is essential for interaction with the NPC (nucleosome core particle); required for autophagy; homologous to Drosophila Enhancer of Polycomb; coding sequence contains length polymorphisms in different strains.	[89]
POL1	*YNL102W*	Catalytic subunit of the DNA polymerase I alpha-primase complex; required for the initiation of DNA replication during mitotic DNA synthesis and premeiotic DNA synthesis.	[90]
POL2	*YNL262W*	Catalytic subunit of DNA polymerase (II) epsilon; a chromosomal DNA replication polymerase that exhibits processivity and proofreading exonuclease activity; participates in leading-strand synthesis during DNA	[17]

		replication; also involved in DNA synthesis during DNA repair; interacts extensively with Mrc1p.	
MEC1	*YBR136W*	Genome integrity checkpoint protein and PI kinase superfamily member; Mec1p and Dun1p function in same pathway to regulate dNTP pools and telomere length; signal transducer required for cell cycle arrest and transcriptional responses to damaged or unreplicated DNA; facilitates replication fork progression and regulates P-body formation under replication stress; promotes interhomolog recombination by phosphorylating Hop1p; associates with shortened, dysfunctional telomeres.	65
RPT4	*YOR259C*	ATPase of the 19S regulatory particle of the 26S proteasome; one of six ATPases of the regulatory particle; involved in degradation of ubiquitinated substrates; contributes preferentially to ERAD; required for spindle pole body duplication; mainly nuclear localization.	91
RPT6	*YGL048C*	ATPase of the 19S regulatory particle of the 26S proteasome; one of six ATPases of the regulatory particle; involved in the degradation of ubiquitinated substrates; bound by ubiquitin-protein ligases Ubr1p and Ufd4p; localized mainly to the nucleus throughout the cell cycle; protein abundance increases in response to DNA replication stress.	91
STN1	*YDR082W*	Telomere end-binding and capping protein; plays a key role with Pol12p in linking telomerase action with completion of lagging strand synthesis, and in a regulatory step required for telomere capping; similar to human Stn1.	92

References (Note S1)

1 Cherry, J. M. *et al.* Saccharomyces Genome Database: the genomics resource of budding yeast. *Nucleic acids research* **40**, D700-705, doi:10.1093/nar/gkr1029 (2012).

2 Zakian, M. A. M. V. A. in *Telomeres* Vol. 45 *Cold Spring Harbor Monograph Series* (ed Vicki Lundblad Titia de Lange, Elizabeth Blackburn) Ch. 10, 261-316 (Cold Spring Harbor Laboratory Press, 2006).

3 Smith, J. J. *et al.* Environment-responsive transcription factors bind subtelomeric elements and regulate gene silencing. *Mol Syst Biol* **7**, 455, doi:10.1038/msb.2010.110 (2011).

4 Neves-Costa, A., Will, W. R., Vetter, A. T., Miller, J. R. & Varga-Weisz, P. The SNF2-family member Fun30 promotes gene silencing in heterochromatic loci. *PloS one* **4**, e8111, doi:10.1371/journal.pone.0008111 (2009).

5 Krogan, N. J. *et al.* The Paf1 complex is required for histone H3 methylation by COMPASS and Dot1p: linking transcriptional elongation to histone methylation. *Mol Cell* **11**, 721-729 (2003).

6 Chang, J. S. & Winston, F. Spt10 and Spt21 are required for transcriptional silencing in Saccharomyces cerevisiae. *Eukaryotic cell* **10**, 118-129, doi:10.1128/EC.00246-10 (2011).

7 Andrulis, E. D. *et al.* Esc1, a nuclear periphery protein required for Sir4-based plasmid anchoring and partitioning. *Mol Cell Biol* **22**, 8292-8301 (2002).

8 Braun, M. A., Costa, P. J., Crisucci, E. M. & Arndt, K. M. Identification of Rkr1, a nuclear RING domain protein with functional connections to chromatin modification in

Saccharomyces cerevisiae. *Molecular and cellular biology* **27**, 2800-2811, doi:10.1128/MCB.01947-06 (2007).
9 Roy, N. & Runge, K. W. The ZDS1 and ZDS2 proteins require the Sir3p component of yeast silent chromatin to enhance the stability of short linear centromeric plasmids. *Chromosoma* **108**, 146-161 (1999).
10 Boulton, S. J. & Jackson, S. P. Components of the Ku-dependent non-homologous end-joining pathway are involved in telomeric length maintenance and telomeric silencing. *EMBO J* **17**, 1819-1828, doi:10.1093/emboj/17.6.1819 (1998).
11 Koch, M. R. & Pillus, L. The glucanosyltransferase Gas1 functions in transcriptional silencing. *Proc Natl Acad Sci U S A* **106**, 11224-11229, doi:10.1073/pnas.0900809106 (2009).
12 Burgess, R. J., Guy, M. P. & Zhang, Z. Fueling transcriptional silencing with Gas1. *Proc Natl Acad Sci U S A* **106**, 10879-10880, doi:10.1073/pnas.0905192106 (2009).
13 Rundlett, S. E. *et al.* HDA1 and RPD3 are members of distinct yeast histone deacetylase complexes that regulate silencing and transcription. *Proc Natl Acad Sci U S A* **93**, 14503-14508 (1996).
14 Brachmann, C. B. *et al.* The SIR2 gene family, conserved from bacteria to humans, functions in silencing, cell cycle progression, and chromosome stability. *Genes Dev* **9**, 2888-2902 (1995).
15 Kaufman, P. D., Cohen, J. L. & Osley, M. A. Hir proteins are required for position-dependent gene silencing in Saccharomyces cerevisiae in the absence of chromatin assembly factor I. *Mol Cell Biol* **18**, 4793-4806 (1998).
16 Dror, V. & Winston, F. The Swi/Snf chromatin remodeling complex is required for ribosomal DNA and telomeric silencing in Saccharomyces cerevisiae. *Mol Cell Biol* **24**, 8227-8235, doi:10.1128/MCB.24.18.8227-8235.2004 (2004).
17 Iida, T. & Araki, H. Noncompetitive counteractions of DNA polymerase epsilon and ISW2/yCHRAC for epigenetic inheritance of telomere position effect in Saccharomyces cerevisiae. *Mol Cell Biol* **24**, 217-227 (2004).
18 Sun, Z. W. & Hampsey, M. A general requirement for the Sin3-Rpd3 histone deacetylase complex in regulating silencing in Saccharomyces cerevisiae. *Genetics* **152**, 921-932 (1999).
19 Meneghini, M. D., Wu, M. & Madhani, H. D. Conserved histone variant H2A.Z protects euchromatin from the ectopic spread of silent heterochromatin. *Cell* **112**, 725-736 (2003).
20 Cuperus, G. & Shore, D. Restoration of silencing in Saccharomyces cerevisiae by tethering of a novel Sir2-interacting protein, Esc8. *Genetics* **162**, 633-645 (2002).
21 Suzuki, Y. & Nishizawa, M. The yeast GAL11 protein is involved in regulation of the structure and the position effect of telomeres. *Mol Cell Biol* **14**, 3791-3799 (1994).
22 Franke, J., Gehlen, J. & Ehrenhofer-Murray, A. E. Hypermethylation of yeast telomerase RNA by the snRNA and snoRNA methyltransferase Tgs1. *J Cell Sci* **121**, 3553-3560, doi:10.1242/jcs.033308 (2008).
23 Pemberton, L. F. & Blobel, G. Characterization of the Wtm proteins, a novel family of Saccharomyces cerevisiae transcriptional modulators with roles in meiotic regulation and silencing. *Mol Cell Biol* **17**, 4830-4841 (1997).
24 Denisenko, O. & Bomsztyk, K. Yeast hnRNP K-like genes are involved in regulation of the telomeric position effect and telomere length. *Mol Cell Biol* **22**, 286-297 (2002).
25 Moehle, E. A., Ryan, C. J., Krogan, N. J., Kress, T. L. & Guthrie, C. The yeast SR-like protein Npl3 links chromatin modification to mRNA processing. *PLoS genetics* **8**, e1003101, doi:10.1371/journal.pgen.1003101 (2012).
26 Kelly, T. J., Qin, S., Gottschling, D. E. & Parthun, M. R. Type B histone acetyltransferase Hat1p participates in telomeric silencing. *Mol Cell Biol* **20**, 7051-7058 (2000).

27 Wang, X., Connelly, J. J., Wang, C. L. & Sternglanz, R. Importance of the Sir3 N terminus and its acetylation for yeast transcriptional silencing. *Genetics* **168**, 547-551, doi:10.1534/genetics.104.028803 (2004).

28 Aparicio, O. M., Billington, B. L. & Gottschling, D. E. Modifiers of position effect are shared between telomeric and silent mating-type loci in S. cerevisiae. *Cell* **66**, 1279-1287 (1991).

29 Ray, A. *et al.* Sir3p phosphorylation by the Slt2p pathway effects redistribution of silencing function and shortened lifespan. *Nat Genet* **33**, 522-526, doi:10.1038/ng1132 (2003).

30 Ivessa, A. S., Zhou, J. Q., Schulz, V. P., Monson, E. K. & Zakian, V. A. Saccharomyces Rrm3p, a 5' to 3' DNA helicase that promotes replication fork progression through telomeric and subtelomeric DNA. *Genes Dev* **16**, 1383-1396, doi:10.1101/gad.982902 (2002).

31 Lew, J. E., Enomoto, S. & Berman, J. Telomere length regulation and telomeric chromatin require the nonsense-mediated mRNA decay pathway. *Mol Cell Biol* **18**, 6121-6130 (1998).

32 Hu, F., Alcasabas, A. A. & Elledge, S. J. Asf1 links Rad53 to control of chromatin assembly. *Genes Dev* **15**, 1061-1066, doi:10.1101/gad.873201 (2001).

33 Smolikov, S., Mazor, Y. & Krauskopf, A. ELG1, a regulator of genome stability, has a role in telomere length regulation and in silencing. *Proc Natl Acad Sci U S A* **101**, 1656-1661, doi:10.1073/pnas.0307796100 (2004).

34 Sandmeier, J. J., Celic, I., Boeke, J. D. & Smith, J. S. Telomeric and rDNA silencing in Saccharomyces cerevisiae are dependent on a nuclear NAD(+) salvage pathway. *Genetics* **160**, 877-889 (2002).

35 Xu, E. Y., Kim, S. & Rivier, D. H. SAS4 and SAS5 are locus-specific regulators of silencing in Saccharomyces cerevisiae. *Genetics* **153**, 25-33 (1999).

36 Stone, E. M. & Pillus, L. Activation of an MAP kinase cascade leads to Sir3p hyperphosphorylation and strengthens transcriptional silencing. *J Cell Biol* **135**, 571-583 (1996).

37 Corda, Y. *et al.* Interaction between Set1p and checkpoint protein Mec3p in DNA repair and telomere functions. *Nat Genet* **21**, 204-208, doi:10.1038/5991 (1999).

38 Wyatt, H. R., Liaw, H., Green, G. R. & Lustig, A. J. Multiple roles for Saccharomyces cerevisiae histone H2A in telomere position effect, Spt phenotypes and double-strand-break repair. *Genetics* **164**, 47-64 (2003).

39 Ng, H. H. *et al.* Lysine methylation within the globular domain of histone H3 by Dot1 is important for telomeric silencing and Sir protein association. *Genes & development* **16**, 1518-1527, doi:10.1101/gad.1001502 (2002).

40 Li, Q. *et al.* The elongator complex interacts with PCNA and modulates transcriptional silencing and sensitivity to DNA damage agents. *PLoS Genet* **5**, e1000684, doi:10.1371/journal.pgen.1000684 (2009).

41 Perrod, S. *et al.* A cytosolic NAD-dependent deacetylase, Hst2p, can modulate nucleolar and telomeric silencing in yeast. *EMBO J* **20**, 197-209, doi:10.1093/emboj/20.1.197 (2001).

42 Power, P., Jeffery, D., Rehman, M. A., Chatterji, A. & Yankulov, K. Sub-telomeric core X and Y' elements in S. cerevisiae suppress extreme variations in gene silencing. *PLoS One* **6**, e17523, doi:10.1371/journal.pone.0017523 (2011).

43 Huang, H., Kahana, A., Gottschling, D. E., Prakash, L. & Liebman, S. W. The ubiquitin-conjugating enzyme Rad6 (Ubc2) is required for silencing in Saccharomyces cerevisiae. *Mol Cell Biol* **17**, 6693-6699 (1997).

44 Liu, Y. *et al.* Structural analysis of Rtt106p reveals a DNA binding role required for heterochromatin silencing. *J Biol Chem* **285**, 4251-4262, doi:10.1074/jbc.M109.055996 (2010).

45 Chi, M. H. & Shore, D. SUM1-1, a dominant suppressor of SIR mutations in Saccharomyces cerevisiae, increases transcriptional silencing at telomeres and HM mating-

type loci and decreases chromosome stability. *Molecular and cellular biology* **16**, 4281-4294, doi:10.1128/MCB.16.8.4281 (1996).
46 Singer, M. S. *et al.* Identification of high-copy disruptors of telomeric silencing in Saccharomyces cerevisiae. *Genetics* **150**, 613-632 (1998).
47 Yu, E. Y. *et al.* Regulation of telomere structure and functions by subunits of the INO80 chromatin remodeling complex. *Molecular and cellular biology* **27**, 5639-5649, doi:10.1128/MCB.00418-07 (2007).
48 Kaufman, P. D., Kobayashi, R. & Stillman, B. Ultraviolet radiation sensitivity and reduction of telomeric silencing in Saccharomyces cerevisiae cells lacking chromatin assembly factor-I. *Genes Dev* **11**, 345-357 (1997).
49 Sekiguchi, T., Hayashi, N., Wang, Y. & Kobayashi, H. Genetic evidence that Ras-like GTPases, Gtr1p, and Gtr2p, are involved in epigenetic control of gene expression in Saccharomyces cerevisiae. *Biochem Biophys Res Commun* **368**, 748-754, doi:10.1016/j.bbrc.2008.01.133 (2008).
50 Suka, N., Luo, K. & Grunstein, M. Sir2p and Sas2p opposingly regulate acetylation of yeast histone H4 lysine16 and spreading of heterochromatin. *Nat Genet* **32**, 378-383, doi:10.1038/ng1017 (2002).
51 Kimura, A., Umehara, T. & Horikoshi, M. Chromosomal gradient of histone acetylation established by Sas2p and Sir2p functions as a shield against gene silencing. *Nat Genet* **32**, 370-377, doi:10.1038/ng993 (2002).
52 Chang, J. S. & Winston, F. Spt10 and Spt21 Are Required for Transcriptional Silencing in Saccharomyces cerevisiae. *Eukaryotic Cell* **10**, 118-129, doi:10.1128/Ec.00246-10 (2011).
53 Le, S., Davis, C., Konopka, J. B. & Sternglanz, R. Two new S-phase-specific genes from Saccharomyces cerevisiae. *Yeast* **13**, 1029-1042, doi:10.1002/(SICI)1097-0061(19970915)13:11<1029::AID-YEA160>3.0.CO;2-1 (1997).
54 Ringel, A. E. *et al.* Yeast Tdh3 (glyceraldehyde 3-phosphate dehydrogenase) is a Sir2-interacting factor that regulates transcriptional silencing and rDNA recombination. *PLoS Genet* **9**, e1003871, doi:10.1371/journal.pgen.1003871 (2013).
55 Pryde, F. E. & Louis, E. J. Limitations of silencing at native yeast telomeres. *EMBO J* **18**, 2538-2550, doi:10.1093/emboj/18.9.2538 (1999).
56 Kyrion, G., Liu, K., Liu, C. & Lustig, A. J. RAP1 and telomere structure regulate telomere position effects in Saccharomyces cerevisiae. *Genes Dev* **7**, 1146-1159 (1993).
57 Clement, M., Deshaies, F., de Repentigny, L. & Belhumeur, P. The nuclear GTPase Gsp1p can affect proper telomeric function through the Sir4 protein in Saccharomyces cerevisiae. *Molecular microbiology* **62**, 453-468, doi:10.1111/j.1365-2958.2006.05374.x (2006).
58 van Welsem, T. *et al.* Synthetic lethal screens identify gene silencing processes in yeast and implicate the acetylated amino terminus of Sir3 in recognition of the nucleosome core. *Mol Cell Biol* **28**, 3861-3872, doi:10.1128/MCB.02050-07 (2008).
59 Mann, R. K. & Grunstein, M. Histone H3 N-terminal mutations allow hyperactivation of the yeast GAL1 gene in vivo. *EMBO J* **11**, 3297-3306 (1992).
60 Thompson, J. S., Ling, X. & Grunstein, M. Histone H3 amino terminus is required for telomeric and silent mating locus repression in yeast. *Nature* **369**, 245-247, doi:10.1038/369245a0 (1994).
61 Zhang, Z., Shibahara, K. & Stillman, B. PCNA connects DNA replication to epigenetic inheritance in yeast. *Nature* **408**, 221-225, doi:10.1038/35041601 (2000).
62 Crotti, L. B. & Basrai, M. A. Functional roles for evolutionarily conserved Spt4p at centromeres and heterochromatin in Saccharomyces cerevisiae. *EMBO J* **23**, 1804-1814, doi:10.1038/sj.emboj.7600161 (2004).
63 Darst, R. P., Garcia, S. N., Koch, M. R. & Pillus, L. Slx5 promotes transcriptional silencing and is required for robust growth in the absence of Sir2. *Molecular and cellular biology* **28**, 1361-1372, doi:10.1128/MCB.01291-07 (2008).

64 Jacobson, S. & Pillus, L. The SAGA subunit Ada2 functions in transcriptional silencing. *Molecular and cellular biology* **29**, 6033-6045, doi:10.1128/MCB.00542-09 (2009).

65 Craven, R. J. & Petes, T. D. Dependence of the regulation of telomere length on the type of subtelomeric repeat in the yeast Saccharomyces cerevisiae. *Genetics* **152**, 1531-1541 (1999).

66 Wood, A. *et al.* Bre1, an E3 ubiquitin ligase required for recruitment and substrate selection of Rad6 at a promoter. *Mol Cell* **11**, 267-274 (2003).

67 Ladurner, A. G., Inouye, C., Jain, R. & Tjian, R. Bromodomains mediate an acetyl-histone encoded antisilencing function at heterochromatin boundaries. *Mol Cell* **11**, 365-376 (2003).

68 Burgess, R. J., Zhou, H., Han, J., Li, Q. & Zhang, Z. The SCFDia2 ubiquitin E3 ligase ubiquitylates Sir4 and functions in transcriptional silencing. *PLoS genetics* **8**, e1002846, doi:10.1371/journal.pgen.1002846 (2012).

69 Liachko, I. & Tye, B. K. Mcm10 is required for the maintenance of transcriptional silencing in Saccharomyces cerevisiae. *Genetics* **171**, 503-515, doi:10.1534/genetics.105.042333 (2005).

70 Liachko, I. & Tye, B. K. Mcm10 mediates the interaction between DNA replication and silencing machineries. *Genetics* **181**, 379-391, doi:10.1534/genetics.108.099101 (2009).

71 Ghosh, S. *et al.* Acetylation of the SUN protein Mps3 by Eco1 regulates its function in nuclear organization. *Mol Biol Cell* **23**, 2546-2559, doi:10.1091/mbc.E11-07-0600 (2012).

72 Hayashi, N. *et al.* Mutations in Ran system affected telomere silencing in Saccharomyces cerevisiae. *Biochem Biophys Res Commun* **363**, 788-794, doi:10.1016/j.bbrc.2007.09.054 (2007).

73 Marsellach, F. X., Huertas, D. & Azorin, F. The multi-KH domain protein of Saccharomyces cerevisiae Scp160p contributes to the regulation of telomeric silencing. *J Biol Chem* **281**, 18227-18235, doi:10.1074/jbc.M601671200 (2006).

74 Lahue, E., Heckathorn, J., Meyer, Z., Smith, J. & Wolfe, C. The Saccharomyces cerevisiae Sub2 protein suppresses heterochromatic silencing at telomeres and subtelomeric genes. *Yeast* **22**, 537-551, doi:10.1002/yea.1231 (2005).

75 Enomoto, S., McCune-Zierath, P. D., Gerami-Nejad, M., Sanders, M. A. & Berman, J. RLF2, a subunit of yeast chromatin assembly factor-I, is required for telomeric chromatin function in vivo. *Genes Dev* **11**, 358-370, doi:10.1101/gad.11.3.358 (1997).

76 Monson, E. K., de Bruin, D. & Zakian, V. A. The yeast Cac1 protein is required for the stable inheritance of transcriptionally repressed chromatin at telomeres. *Proceedings of the National Academy of Sciences of the United States of America* **94**, 13081-13086, doi:10.1073/pnas.94.24.13081 (1997).

77 Poveda, A. *et al.* Hif1 is a component of yeast histone acetyltransferase B, a complex mainly localized in the nucleus. *J Biol Chem* **279**, 16033-16043, doi:10.1074/jbc.M314228200 (2004).

78 Ai, X. & Parthun, M. R. The nuclear Hat1p/Hat2p complex: a molecular link between type B histone acetyltransferases and chromatin assembly. *Mol Cell* **14**, 195-205, doi:10.1016/s1097-2765(04)00184-4 (2004).

79 Rehman, M. A. *et al.* Differential requirement of DNA replication factors for subtelomeric ARS consensus sequence protosilencers in Saccharomyces cerevisiae. *Genetics* **174**, 1801-1810, doi:10.1534/genetics.106.063446 (2006).

80 Huang, S. *et al.* Rtt106p is a histone chaperone involved in heterochromatin-mediated silencing. *Proceedings of the National Academy of Sciences of the United States of America* **102**, 13410-13415, doi:10.1073/pnas.0506176102 (2005).

81 Moretti, P., Freeman, K., Coodly, L. & Shore, D. Evidence that a complex of SIR proteins interacts with the silencer and telomere-binding protein RAP1. *Genes Dev* **8**, 2257-2269, doi:10.1101/gad.8.19.2257 (1994).

82 Luo, K., Vega-Palas, M. A. & Grunstein, M. Rap1-Sir4 binding independent of other Sir, yKu, or histone interactions initiates the assembly of telomeric heterochromatin in yeast. *Genes Dev* **16**, 1528-1539, doi:10.1101/gad.988802 (2002).

83 Nagy, P. L., Griesenbeck, J., Kornberg, R. D. & Cleary, M. L. A trithorax-group complex purified from Saccharomyces cerevisiae is required for methylation of histone H3. *Proceedings of the National Academy of Sciences of the United States of America* **99**, 90-94, doi:10.1073/pnas.221596698 (2002).

84 Krogan, N. J. *et al.* COMPASS, a histone H3 (Lysine 4) methyltransferase required for telomeric silencing of gene expression. *J Biol Chem* **277**, 10753-10755, doi:10.1074/jbc.C200023200 (2002).

85 Nislow, C., Ray, E. & Pillus, L. SET1, a yeast member of the trithorax family, functions in transcriptional silencing and diverse cellular processes. *Mol Biol Cell* **8**, 2421-2436, doi:10.1091/mbc.8.12.2421 (1997).

86 Emre, N. C. *et al.* Maintenance of low histone ubiquitylation by Ubp10 correlates with telomere-proximal Sir2 association and gene silencing. *Mol Cell* **17**, 585-594, doi:10.1016/j.molcel.2005.01.007 (2005).

87 Larin, M. L. *et al.* Competition between Heterochromatic Loci Allows the Abundance of the Silencing Protein, Sir4, to Regulate de novo Assembly of Heterochromatin. *PLoS genetics* **11**, e1005425, doi:10.1371/journal.pgen.1005425 (2015).

88 Fox, C. A., Ehrenhofer-Murray, A. E., Loo, S. & Rine, J. The origin recognition complex, SIR1, and the S phase requirement for silencing. *Science* **276**, 1547-1551 (1997).

89 Boudreault, A. A. *et al.* Yeast enhancer of polycomb defines global Esa1-dependent acetylation of chromatin. *Genes Dev* **17**, 1415-1428, doi:10.1101/gad.1056603 (2003).

90 Adams Martin, A., Dionne, I., Wellinger, R. J. & Holm, C. The function of DNA polymerase alpha at telomeric G tails is important for telomere homeostasis. *Molecular and cellular biology* **20**, 786-796, doi:10.1128/MCB.20.3.786-796.2000 (2000).

91 Ezhkova, E. & Tansey, W. P. Proteasomal ATPases link ubiquitylation of histone H2B to methylation of histone H3. *Mol Cell* **13**, 435-442, doi:10.1016/s1097-2765(04)00026-7 (2004).

92 Dahlseid, J. N. *et al.* mRNAs encoding telomerase components and regulators are controlled by UPF genes in Saccharomyces cerevisiae. *Eukaryot Cell* **2**, 134-142 (2003).
