# Supplementary material for: Systematic profiling of subtelomeric silencing factors in budding yeast
Source: G3 (Bethesda). 2023 Jul 11;13(10):jkad153. doi: 10.1093/g3journal/jkad153 (PMC10542202; doi:10.1093/g3journal/jkad153)
Supplement: jkad153_Supplementary_Data [file jkad153_supplementary_data.zip › Supplemental_Material_Legends_G3-2022-403752.docx]

**SUPPLEMENTARY MATERIAL**

**Note S1.** List of genes previously associated with telomeric silencing in *Saccharomyces cerevisiae*

**Table S1.** List of strains used in this study

**Table S2.** List of primers used in this study

**Table S3.** List of genes used for *kappa* statistical analysis

**Table S4.** Features of different subtelomeric loci and silencing analysis

**Figure S1.** Microscopy analysis of GFP expression at different loci

**Figure S2.** Insertion of the *URA3-*GFP does not disrupt nucleosome positioning at the *YFR057W* promoter

**Figure S3.** *Si scores* are highly correlated between screens done independently for the same or for different subtelomeric loci

**Figure S4.** Replicability of Si scores obtained for a selected set of mutants

**Figure S5.** 5-FOA growth assays are highly reproducible

**Dataset S1.** Raw data of subtelomeric silencing screens *COS12* and *YFR057W* by flow cytometry (XLS)
