# Supplementary material for: Systematic profiling of subtelomeric silencing factors in budding yeast
Source: G3 (Bethesda). 2023 Jul 11;13(10):jkad153. doi: 10.1093/g3journal/jkad153 (PMC10542202; doi:10.1093/g3journal/jkad153)
Supplement: jkad153_Supplementary_Data [file jkad153_supplementary_data.zip › Table_S1_G3-2022-403752.pdf]

Supplementary Table S1. Features of different subtelomeric loci and silencing analysis.

Locus	Telomere	End type [a]	Direction to telomere [b]	ATG to core X (bp)	ATG to telomere (bp)	ORF no. from core X	Silencing strength [c]	GFP signal [d]
YFR057W	VI-R	X	→ *////*	670	1,100	1	-	350.2
COS1	XIV-L	X-Y'	← *////*	903	8,330	1	+++	300.7
COS2	II-R	X	← *////*	899	1,705	1	+	331.5
COS4	VI-L	X-Y'	← *////*	895	6,426	1	+	313.5
COS5	X-R	X	← *////*	902	1,752	1	+	376.8
COS7	IV-L	X	← *////*	897	1,802	1	++	231.2
COS8	VIII-L	X-Y'	← *////*	895	6,401	1	++++	230.0
COS10	XIV-R	X	→ *////*	3,362	4,417	2	+	352.4
COS12	VII-L	X	← *////*	2,010	2,790	1	+++	177.7

a. End-type indicates the presence of subtelomeric conserved elements X and Y’ in the chromosome.
b. Coding direction of the ORF relative to telomere (*////*).
c. Silencing strength estimated by observed growth in 5-FOA. This study.
d. GFP expression measured by flow cytometry. GFP signal is defined as mean GFP signal minus mean GFP background fluorescence in the *ura3-* parental strain with no insertion (158.9 a.u.). This study.
