# Supplementary material for: Systematic profiling of subtelomeric silencing factors in budding yeast
Source: G3 (Bethesda). 2023 Jul 11;13(10):jkad153. doi: 10.1093/g3journal/jkad153 (PMC10542202; doi:10.1093/g3journal/jkad153)
Supplement: jkad153_Supplementary_Data [file jkad153_supplementary_data.zip › Table_S2_G3-2022-403752.pdf]

Supplementary Table S2. Genes used for kappa analysis

Hits with an FDR <10% in *COS12* or *YFR057W* screenings (this study)

Gene	Description
COR1	Core subunit of the ubiquinol-cytochrome c reductase complex
RPS8A	Protein component of the small (40S) ribosomal subunit
NUP170	Subunit of inner ring of nuclear pore complex (NPC)
FUR4	Plasma membrane localized uracil permease
SCO1	Copper-binding protein of mitochondrial inner membrane
UBP14	Ubiquitin-specific protease
SIF2	WD40 repeat-containing subunit of Set3C histone deacetylase complex
RPL21A	Ribosomal 60S subunit protein L21A
PDB1	E1 beta subunit of the pyruvate dehydrogenase (PDH) complex
REI1	Cytoplasmic pre-60S factor
RIF1	Protein involved in control of DNA replication
YCR006C	Putative protein of unknown function
YDL068W	Dubious open reading frame
CBS1	Mitochondrial translational activator of the COB mRNA
MSS2	Peripherally bound inner membrane protein of the mitochondrial matrix
IWR1	RNA polymerase II transport factor, conserved from yeast to humans
CRD1	Cardiolipin synthase
BUD30	Dubious open reading frame
KCS1	Inositol hexakisphosphate and inositol heptakisphosphate kinase
PET100	Chaperone that facilitates the assembly of cytochrome c oxidase
VPS41	Subunit of the HOPS endocytic tethering complex
MRX14	Putative mitochondrial ribosomal protein of the large subunit
INO2	Transcription factor
VPS64	Protein required for cytoplasm to vacuole targeting of proteins
MRPL35	Mitochondrial ribosomal protein of the large subunit
MSN5	Karyopherin
MRP1	Mitochondrial ribosomal protein of the small subunit
BCS1	Protein translocase and chaperone required for Complex III assembly
ATP17	Subunit f of the F0 sector of mitochondrial F1F0 ATP synthase
SHE9	Protein required for normal mitochondrial morphology
SIZ1	SUMO E3 ligase
ERD1	Predicted membrane protein required for lumenal ER protein retention
SNF1	AMP-activated S/T protein kinase
SPF1	P-type ATPase, ion transporter of the ER membrane
RPL12A	Ribosomal 60S subunit protein L12A
CIN8	Bipolar kinesin motor protein
GLN3	Transcriptional activator in nitrogen catabolite repression system
RSM18	Mitochondrial ribosomal protein of the small subunit
PET117	Assembly factor that couples heme a synthesis to complex IV assembly
CEM1	Mitochondrial beta-keto-acyl synthase
SWI4	DNA binding component of the SBF complex (Swi4p-Swi6p)
RTR1	Dual specificity protein phosphatase
OXA1	Mitochondrial inner membrane insertase
CHD1	Chromatin remodeler that regulates various aspects of transcription
PDA1	E1 alpha subunit of the pyruvate dehydrogenase (PDH) complex
YGL024W	Dubious open reading frame

SNF4	Activating gamma subunit of the AMP-activated Snf1p kinase complex
ITC1	Subunit of ATP-dependent Isw2p-Itc1p chromatin remodeling complex
MRF1	Mitochondrial translation release factor
COX18	Protein required for membrane insertion of C-terminus of Cox2p
ENV11	Protein proposed to be involved in vacuolar functions
SHY1	Mitochondrial inner membrane protein required for complex IV assembly
CHO2	Phosphatidylethanolamine methyltransferase (PEMT)
PDX1	E3-binding protein of the mitochondrial pyruvate dehydrogenase complex
RPL8A	Ribosomal 60S subunit protein L8A
CBP2	Required for splicing of the group I intron bI5 of the COB pre-mRNA
SSZ1	Hsp70 protein that interacts with Zuo1p (a DnaJ homolog)
IRE1	Serine-threonine kinase and endoribonuclease
PTH1	One of two mitochondrially-localized peptidyl-tRNA hydrolases
RPN10	Proteasome polyubiquitin receptor
CST6	Basic leucine zipper (bZIP) transcription factor from ATF/CREB family
MET18	Component of cytosolic iron-sulfur protein assembly (CIA) machinery
MRPL8	Mitochondrial ribosomal protein of the large subunit
MRPL49	Mitochondrial ribosomal protein of the large subunit
TRK1	Component of the Trk1p-Trk2p potassium transport system
INO1	Inositol-3-phosphate synthase
SET2	Histone methyltransferase with a role in transcriptional elongation
YJL169W	Dubious open reading frame
YJL175W	Dubious open reading frame unlikely to encode a functional protein
MNN11	Subunit of a Golgi mannosyltransferase complex
ACO2	Putative mitochondrial aconitase isozyme
RCY1	F-box protein involved in recycling endocytosed proteins
CBP1	Mitochondrial protein, regulator of COB mRNA stability and translation
TOR1	PIK-related protein kinase and rapamycin target
ADO1	Adenosine kinase
HOM6	Homoserine dehydrogenase (L-homoserine:NADP oxidoreductase)
MRP17	Mitochondrial ribosomal protein of the small subunit
NFU1	Protein involved in Fe-S cluster transfer to mitochondrial clients
NUP120	Subunit of the Nup84p subcomplex of the nuclear pore complex (NPC)
CYT2	Cytochrome c1 heme lyase
OCT1	Mitochondrial intermediate peptidase
ZRT3	Vacuolar membrane zinc transporter
SAC1	Phosphatidylinositol phosphate phosphatase
URA1	Dihydroorotate dehydrogenase
UBR2	Cytoplasmic ubiquitin-protein ligase (E3)
PET309	Specific translational activator for the COX1 mRNA
HOG1	Mitogen-activated protein kinase involved in osmoregulation
ACE2	Transcription factor required for septum destruction after cytokinesis
YKE2	Subunit of the heterohexameric Gim/prefoldin protein complex
MSS51	Specific translational activator for the mitochondrial COX1 mRNA
QRI5	Mitochondrial inner membrane protein
CDC73	Component of the Paf1p complex
ERG6	Delta(24)-sterol C-methyltransferase
TSA1	Thioredoxin peroxidase
URA5	Major orotate phosphoribosyltransferase (OPRTase) isozyme
PML39	Protein required for nuclear retention of unspliced pre-mRNAs
SUB1	Transcriptional regulator
FET3	Ferro-O2-oxidoreductase

ARG7	Mitochondrial ornithine acetyltransferase
MUB1	MYND domain-containing protein
YKU80	Subunit of telomeric Ku complex (Yku70p-Yku80p)
RPL13B	Ribosomal 60S subunit protein L13B
SPT21	Protein with a role in transcriptional silencing
HFA1	Mitochondrial acetyl-coenzyme A carboxylase
MTF1	Mitochondrial RNA polymerase specificity factor
YKU70	Subunit of the telomeric Ku complex (Yku70p-Yku80p)
HER2	Subunit of the trimeric GatFAB AmidoTransferase(AdT) complex
LAT1	Dihydrolipoamide acetyltransferase component (E2) of the PDC
AAH1	Adenine deaminase (adenine aminohydrolase)
YNL184C	Protein of unknown function
WHI3	RNA binding protein that modulates mRNA stability
RRG9	Protein of unknown function
IES2	Protein that associates with the INO80 chromatin remodeling complex
PET494	Mitochondrial translational activator specific for the COX3 mRNA
POP2	Subunit of Ccr4-Not complex that mediates 3' to 5' mRNA deadenylation
PHO80	Cyclin
YOL050C	Dubious open reading frame
VAM10	Protein involved in vacuole morphogenesis
VAM3	Syntaxin-like vacuolar t-SNARE
LEO1	Component of the Paf1 complex
CAT5	Protein required for ubiquinone (Coenzyme Q) biosynthesis
ARP8	Nuclear actin-related protein involved in chromatin remodeling
ELG1	Subunit of an alternative replication factor C complex
GEP3	Protein required for mitochondrial ribosome small subunit biogenesis
MCT1	Predicted malonyl-CoA:ACP transferase
SSP2	Sporulation specific protein that localizes to the spore wall
ENV9	Conserved oxidoreductase involved in lipid droplet morphology
VPH1	Subunit a of the vacuolar-ATPase V0 domain
ISW2	ATP-dependent DNA translocase involved in chromatin remodeling
LGE1	Protein involved in histone H2B ubiquitination
ELP4	Subunit of hexameric RecA-like ATPase Elp456 Elongator subcomplex
YPL102C	Dubious open reading frame
MRP51	Mitochondrial ribosomal protein of the small subunit
ISU1	Conserved protein of the mitochondrial matrix
POC4	Component of a heterodimeric Poc4p-Irc25p chaperone
COX10	Heme A: farnesyltransferase
ATG41	Protein of unknown function
FUM1	Fumarase
UBA3	Protein that activates Rub1p (NEDD8) before neddylation
MRPL51	Mitochondrial ribosomal protein of the large subunit
YPR172W	Protein of unknown function

Genes affecting TPE, from literature (Supporting Note S1)

Gene	Description
FUN30	Snf2p family member with ATP-dependent chromatin remodeling activity
OAF1	Oleate-activated transcription factor
SWD1	Subunit of the COMPASS (Set1C) complex

HTA2	Histone H2A
HIR1	Subunit of the HIR complex
FUS3	Mitogen-activated serine/threonine protein kinase involved in mating
SAS3	Histone acetyltransferase catalytic subunit of NuA3 complex
PKC1	Protein serine/threonine kinase
HHF1	Histone H4
HHT1	Histone H3
ORC2	Subunit of the origin recognition complex (ORC)
POL30	Proliferating cell nuclear antigen (PCNA)
MEC1	Genome integrity checkpoint protein and PI kinase superfamily member
SWD3	Essential subunit of the COMPASS (Set1C) complex
MSI1	Subunit of chromatin assembly factor I (CAF-1)
MCM7	Component of the Mcm2-7 hexameric helicase complex
PBP2	RNA binding protein
SHG1	Subunit of the COMPASS (Set1C) complex
RIF1	Protein involved in control of DNA replication
DPB3	Third-largest subunit of DNA polymerase II (DNA polymerase epsilon)
MRC1	S-phase checkpoint protein required for DNA replication
SLX5	Subunit of the Slx5-Slx8 SUMO-targeted Ub ligase (STUbL) complex
NAT1	Subunit of protein N-terminal acetyltransferase NatA
SIR2	Conserved NAD+ dependent histone deacetylase of the Sirtuin family
BRE1	E3 ubiquitin ligase
SUB2	Component of the TREX complex required for nuclear mRNA export
STE7	Signal transducing MAP kinase kinase
STN1	Telomere end-binding and capping protein
DPB4	Subunit of DNA pol epsilon and of ISW2 chromatin accessibility complex
SAS4	Subunit of the SAS complex (Sas2p, Sas4p, Sas5p)
HST4	NAD(+)-dependent protein deacetylase
ADR1	Carbon source-responsive zinc-finger transcription factor
HTA1	Histone H2A
SIR4	SIR protein involved in assembly of silent chromatin domains
SUM1	Transcriptional repressor that regulates middle-sporulation genes
NPL3	RNA-binding protein
DOT1	Nucleosomal histone H3-Lys79 methylase
ADA2	Transcription coactivator
SDC1	Subunit of the COMPASS (Set1C) complex
MCM3	Protein involved in DNA replication
HAT2	Subunit of the Hat1p-Hat2p histone acetyltransferase complex
DOT6	Protein involved in rRNA and ribosome biogenesis
SCS2	Integral ER membrane protein, regulates phospholipid metabolism
EPL1	Subunit of NuA4, an essential histone H4/H2A acetyltransferase complex
PNC1	Nicotinamidase that converts nicotinamide to nicotinic acid
RPT6	ATPase of the 19S regulatory particle of the 26S proteasome
RAD6	Ubiquitin-conjugating enzyme (E2)
ITC1	Subunit of ATP-dependent Isw2p-Itc1p chromatin remodeling complex
INO80	Nucleosome spacing factor

RTF1	Subunit of RNAPII-associated chromatin remodeling Paf1 complex
SPT4	Spt4p/5p (DSIF) transcription elongation factor complex subunit
TDH3	Glyceraldehyde-3-phosphate dehydrogenase (GAPDH), isozyme 3
ARD1	Subunit of protein N-terminal acetyltransferase NatA
SLT2	Serine/threonine MAP kinase
RRM3	DNA helicase involved in rDNA replication and Ty1 transposition
NMD2	Protein involved in the nonsense-mediated mRNA decay (NMD) pathway
STE12	Transcription factor that is activated by a MAPK signaling cascade
SET1	Histone methyltransferase, subunit of the COMPASS (Set1C) complex
DOT5	Nuclear thiol peroxidase
MCM10	Essential chromatin-associated protein
YAP5	Basic leucine zipper (bZIP) iron-sensing transcription factor
MPS3	Nuclear envelope protein
DLS1	Subunit of ISW2/yCHRAC chromatin accessibility complex
SCP160	Essential RNA-binding G protein effector of mating response pathway
BCK1	MAPKKK acting in the protein kinase C signaling pathway
GZF3	GATA zinc finger protein
ASF1	Nucleosome assembly factor
SPT10	Histone H3 acetylase with a role in transcriptional regulation
POL32	Third subunit of DNA polymerase delta
MOG1	Conserved nuclear protein that interacts with GTP-Gsp1p
SWD2	Subunit of the COMPASS (Set1C) histone H3K4 methyltransferase complex
PHD1	Transcriptional activator that enhances pseudohyphal growth
MSN4	Stress-responsive transcriptional activator
ABF1	DNA binding protein with possible chromatin-reorganizing activity
SIR1	Protein involved in silencing at mating-type loci HML and HMR
HIF1	Non-essential component of the HAT-B histone acetyltransferase complex
BRE2	Subunit of COMPASS (Set1C) complex
IES3	Subunit of the INO80 chromatin remodeling complex
IFH1	Coactivator, regulates transcription of ribosomal protein (RP) genes
MCM5	Component of the Mcm2-7 hexameric helicase complex
MEC3	DNA damage and meiotic pachytene checkpoint protein
STE11	Signal transducing MEK kinase
BDF1	BET family transcriptional regulator
SIR3	Silencing protein
RIF2	Protein that binds to the Rap1p C-terminus
CAC2	Subunit of chromatin assembly factor I (CAF-1), with Rlf2p and Msi1p
ZDS2	Protein with a role in regulating Swe1p-dependent polarized growth
GTR1	Subunit of a TORC1-stimulating GTPase and the EGO/GSE complex
YKU80	Subunit of telomeric Ku complex (Yku70p-Yku80p)
SAS2	Histone acetyltransferase (HAT) catalytic subunit of the SAS complex
SPT21	Protein with a role in transcriptional silencing
ESC1	Protein involved in telomeric silencing
RNA1	GTPase activating protein (GAP) for Gsp1p
RKR1	RING domain E3 ubiquitin ligase
SAP30	Component of Rpd3L histone deacetylase complex

ZDS1	Protein with a role in regulating Swe1p-dependent polarized growth
YKU70	Subunit of the telomeric Ku complex (Yku70p-Yku80p)
GAS1	Beta-1,3-glucanosyltransferase required for cell wall assembly
HHF2	Histone H4
HHT2	Histone H3
POL1	Catalytic subunit of the DNA polymerase I alpha-primase complex
YAF9	Subunit of NuA4 histone H4 acetyltransferase and SWR1 complexes
UBP10	Ubiquitin-specific protease, deubiquitinates Ub-protein moieties
RTT106	Histone chaperone
RAP1	Essential DNA-binding transcription regulator that binds many loci
ORC5	Subunit of the origin recognition complex (ORC)
POL2	Catalytic subunit of DNA polymerase (II) epsilon
RPD3	Histone deacetylase, component of both the Rpd3S and Rpd3L complexes
SIN3	Component of both the Rpd3S and Rpd3L histone deacetylase complexes
HTZ1	Histone variant H2AZ
ESC8	Protein involved in telomeric and mating-type locus silencing
GAL11	Subunit of the RNA polymerase II mediator complex
HST3	Sirtuin, histone H3 deacetylase
HIR2	Subunit of HIR nucleosome assembly complex
DIA2	Origin-binding F-box protein
ELG1	Subunit of an alternative replication factor C complex
NPT1	Nicotinate phosphoribosyltransferase
SAS5	Subunit of the SAS complex (Sas2p, Sas4p, Sas5p)
WTM2	Transcriptional modulator
WTM1	Transcriptional modulator
MKK1	MAPKK involved in the protein kinase C signaling pathway
RPT4	ATPase of the 19S regulatory particle of the 26S proteasome
SNF2	Catalytic subunit of the SWI/SNF chromatin remodeling complex
ISW2	ATP-dependent DNA translocase involved in chromatin remodeling
HAT1	Catalytic subunit of the Hat1p-Hat2p histone acetyltransferase complex
HST2	Cytoplasmic NAD(+)-dependent protein deacetylase
ELP3	Subunit of Elongator complex
SPP1	Subunit of COMPASS (Set1C)
UME1	Component of both the Rpd3S and Rpd3L histone deacetylase complexes
TGS1	Trimethyl guanosine synthase, conserved nucleolar methyl transferase
RLF2	Largest subunit (p90) of the Chromatin Assembly Complex (CAF-1)
ROX1	Heme-dependent repressor of hypoxic genes
