# Supplementary material for: Systematic profiling of subtelomeric silencing factors in budding yeast
Source: G3 (Bethesda). 2023 Jul 11;13(10):jkad153. doi: 10.1093/g3journal/jkad153 (PMC10542202; doi:10.1093/g3journal/jkad153)
Supplement: jkad153_Supplementary_Data [file jkad153_supplementary_data.zip › Table_S3_G3-2022-403752.pdf]

Supplementary Table S3. List of strains used in this study

Strain	Background / Parental	Genotype	Resistance
BY4741	SC288c	MATa, *his3Δ1, leu2Δ0, ura3Δ0, met15Δ0*	
Y8205	SC288c	MATα, *his3Δ1, leu2Δ0, ura3Δ0, can1Δ::STE2pr-Sp_his5, lyp1Δ::STE3pr-LEU2*	
*geneΔ::kan*MX4	BY4741	MATa, *his3Δ1, leu2Δ0, ura3Δ0, met15Δ0.* *Yeast deletion collection strains (Open Biosystems)*	$G418^R$
sir3Δ	BY4741	*sir3Δ::KanMX*	$G418^R$
SCA111 *sir3Δ*	Y8205	*sir3Δ::KanMx*	$G418^R$
SCA52	Y8205	*hoΔ::TDH3*$_p$*-mCherry-ADH1*$_{ter}$*-TEF*$_p$*-NAT*^{R}*-TEF*$_{ter}$	NAT^R
SCA89	Y8205	*hoΔ::TDH3*$_p$*-BFP-ADH1*$_{ter}$*-TEF*$_p$*-NAT*^{R}*-TEF*$_{ter}$	
SCA58	SCA52	*yfr057wΔ::URA3-GFP,* *hoΔ::TDH3*$_p$*-mCherry-ADH1*$_{ter}$*-TEF*$_p$*-NAT*^{R}*-TEF*$_{ter}$	NAT^R
SCA62	SCA52	*cos12Δ::URA3-GFP,* *hoΔ::TDH3*$_p$*-mCherry-ADH1*$_{ter}$*-TEF*$_p$*-NAT*^{R}*-TEF*$_{ter}$	NAT^R
SCA103	SCA52	*CUP9:URA3-GFP* *hoΔ::TDH3*$_p$*-mCherry-ADH1*$_{ter}$*-TEF*$_p$*-NAT*^{R}*-TEF*$_{ter}$	NAT^R
SCA91	SCA89	*yfr057wΔ::URA3-GFP,* *hoΔ::TDH3p-BFP-ADH1ter-TEFp-NAT*^{R}*-TEFter*	NAT^R
SCA93	SCA89	*cos12Δ::URA3-GFP,* *hoΔ::TDH3*$_p$*-BFP-ADH1*$_{ter}$*-TEF*$_p$*-NAT*^{R}*-TEF*$_{ter}$	NAT^R
SCA107	SCA89	*CUP9:URA3-GFP* *hoΔ::TDH3*$_p$*-BFP-ADH1*$_{ter}$*-TEF*$_p$*-NAT*^{R}*-TEF*$_{ter}$	NAT^R
SCA114	SCA58	*yfr057wΔ::URA3-GFP, sir3Δ::KanMx,* *hoΔ::TDH3*$_p$*-mCherry-ADH1*$_{ter}$*-TEF*$_p$*-NAT*^{R}*-TEF*$_{ter}$	NAT^R $G418^R$
SCA115	SCA62	*cos12Δ::URA3-GFP, sir3Δ::KanMx,* *hoΔ::TDH3*$_p$*-mCherry-ADH1*$_{ter}$*-TEF*$_p$*-NAT*^{R}*-TEF*$_{ter}$	NAT^R $G418^R$
SCA119	SCA91	*yfr057wΔ::URA3-GFP, sir3Δ::KanMx,* *hoΔ::TDH3*$_p$*-BFP-ADH1*$_{ter}$*-TEF*$_p$*-NAT*^{R}*-TEF*$_{ter}$	NAT^R $G418^R$
SCA121	SCA93	*cos12Δ::URA3-GFP, sir3Δ::KanMx,* *hoΔ::TDH3*$_p$*-BFP-ADH1*$_{ter}$*-TEF*$_p$*-NAT*^{R}*-TEF*$_{ter}$	NAT^R $G418^R$
SCA122	SCA103	*CUP9:URA3-GFP, sir3Δ::KanMx,* *hoΔ::TDH3*$_p$*-mCherry-ADH1*$_{ter}$*-TEF*$_p$*-NAT*^{R}*-TEF*$_{ter}$	NAT^R $G418^R$
cos1Δ	SCA52	*cos1Δ::URA3-GFP,* *hoΔ::TDH3*$_p$*-mCherry-ADH1*$_{ter}$*-TEF*$_p$*-NAT*^{R}*-TEF*$_{ter}$	NAT^R
cos2Δ	SCA52	*cos2Δ::URA3-GFP,* *hoΔ::TDH3*$_p$*-mCherry-ADH1*$_{ter}$*-TEF*$_p$*-NAT*^{R}*-TEF*$_{ter}$	NAT^R
cos4Δ	SCA52	*cos4Δ::URA3-GFP,* *hoΔ::TDH3*$_p$*-mCherry-ADH1*$_{ter}$*-TEF*$_p$*-NAT*^{R}*-TEF*$_{ter}$	NAT^R
cos5Δ	SCA52	*cos5Δ::URA3-GFP,* *hoΔ::TDH3*$_p$*-mCherry-ADH1*$_{ter}$*-TEF*$_p$*-NAT*^{R}*-TEF*$_{ter}$	NAT^R
cos7Δ	SCA52	*cos7Δ::URA3-GFP,* *hoΔ::TDH3*$_p$*-mCherry-ADH1*$_{ter}$*-TEF*$_p$*-NAT*^{R}*-TEF*$_{ter}$	NAT^R
cos10Δ	SCA52	*cos10Δ::URA3-GFP,* *hoΔ::TDH3*$_p$*-mCherry-ADH1*$_{ter}$*-TEF*$_p$*-NAT*^{R}*-TEF*$_{ter}$	NAT^R
