# Supplementary material for: Systematic profiling of subtelomeric silencing factors in budding yeast
Source: G3 (Bethesda). 2023 Jul 11;13(10):jkad153. doi: 10.1093/g3journal/jkad153 (PMC10542202; doi:10.1093/g3journal/jkad153)
Supplement: jkad153_Supplementary_Data [file jkad153_supplementary_data.zip › Table_S4_G3-2022-403752.pdf]

Supplementary Table S4. List of primers used in this study

Primers for construction of *URA3*-GFP reporter

Primer	Sequence
PURA3-Fw +F1	CCAGCTGAAGCTTCGTACGCacagcttttcaattcaattcatcat
URA3-Rv fus GFP	AACCCGGGGATCCGTCGACCgttttgctggccgcatcttctcaaa
GFP-Fw fus URA3	GGTCGACGGATCCCCGGGTTaattaacagtaaaggagaagaactt
GFP-ADH1 terRv+R2	TCGATGAATTCGAGCTCGTTatattaccctgttatccctagcgga

Primers for construction of *URA3*-GFP reporter strains

Primer	Sequence
YFR057Wat-40FwDel	CAAATGTGAAATAGTAGCAACAAAAGGTCATAAATCTTCACCAGCTGAAGCTTCGTACGC
YFR057Wat+40RvDel	CTCCGACAGTTATATCAAAAAAGGCTAAATATAGGAGGCATCGATGAATTCGAGCTCGTT
COS12at-40FwDel	AACTGCAAAATAATGTCTCCTGAACTACATCGCCATAGGCCCAGCTGAAGCTTCGTACGC
COS12at+40RvDel	TTTTTGGTCTTTAAGAATAGTATATCAATATCTTTACCAATCGATGAATTCGAGCTCGTT
COS8at-40FwDel	CGAATAAAAAACCCCTCGAACTGCCATCTCACTACCGAAACCAGCTGAAGCTTCGTACGC
COS8at+40RvDel	GAACAAATGTTCCTTTTCCAAAATATACTATGTGTAGTTGTCGATGAATTCGAGCTCGTT
CUP9at-1098-Fw-Del	TAGTAAGCTTGTAAAGGAATGCACGTATTAGAAAGTGAGCTCGATGAATTCGAGCTCGTT
CUP9at-940-Rv-Del	CGAAAAGCATATGTATAATAGGCACCGACATTTGTGCGGCCCAGCTGAAGCTTCGTACGC
Diag URA3at 120 Rv	GGTACGAACATCCAATGAAGCACAC
Diag GFPat441 Fw	ATTATCAACAAAATACTCCAATTGG
DiagYFR057Wat-170Fw	ATTTAAAAGTGCTAAAGGAATCCCC
DiagYFR057Wat+179Rv	ATCCAATTTTGACATATCCTTCACG
DiagCOS12at-151Fw	TATAGCGACTGTGATATAATGCGCG
DiagCOS12at+174Rv	CAAGTAATCTCATGATATGGCGACC
DiagCOS8at-154Fw	TTGTTTCCCTTTTTCTATCACGAGG
DiagCOS8at+274Rv	ATGACACCATGGCAGTATTCTTACC
DiagCUP9at-1226Fw	AGAGGAGGAGAAAGAGGGAGCTATC
DiagCUP9at-794Rv	TTGGAAGTGTGGACTAGGTCAAGCC
COS2-del2Fw	TGTAATGGTAAAAAGTAGTCTCGTTTTGGTAATTCGCAGCCCAGCTGAAGCTTCGTACGC
COS2-del2Rv	AAGAGAATGAACTTAAAAATGAGAAGAGTGTAGATGTATTTCGATGAATTCGAGCTCGTT
COS7-del4Fw	ATGTACCCGCATTAAAGTTTTGTAAATTCGTTATTACGATTATTGCCAGCTGAAGCTTCGTACGC
COS7-del4Rv	TGAGCTGGTAAGATGAGGATATTGTAGAATAGCATTTTTGCTCTTATGTCGATGAATTCGAGCTCGTT
COS4-del7Fw	CGAATAAAAAACCCCTCGAACTGCCATCTCATTACCGAAACCAGCTGAAGCTTCGTACGC
COS4-del7Rv	AAACTACAAAATATATAAATTGAAAAAGTTGTATTTATCTTCGATGAATTCGAGCTCGTT
COS6-del8Fw	ATCGGAAATGAAATGGATGCCCTTTTCGAATAGTAAAGTTTCTGTGCCCCAGCTGAAGCTTCGTACGC
COS6-del8Rv	CTACTGATGCGTGGAGCAAGCCACGCCATGTGATTAACCTTGCTTGTCGATGAATTCGAGCTCGTT
COS5-del11Fw	CGCAAGAGATATAAAAATTTATAATAAACGTACGTTATGCCCCAGCTGAAGCTTCGTACGC
COS5-del11Rv	CGCATTAAAGTTTTGTAAATTCGTTATTACGATTATCGTCGATGAATTCGAGCTCGTT
COS9-del12Fw	GATACGCCCGCTTGGCGGCTTTTTCTTTCCGACTATATAACCAGCTGAAGCTTCGTACGC
COS9-del12Rv	TAAACTACACGATCTATAAATTGAAAAGGCATGCTTGTTCTCGATGAATTCGAGCTCGTT
COS3-del14Fw	ATACAGAATCTCAAAACAAGCGGGAGAAGTGCTAATTACCCAGCCAGCTGAAGCTTCGTACGC
COS3-del14Rv	ATTCGCATTTTCCTATAAAGCTCTACCTGGGACTGCATCGATGAATTCGAGCTCGTT
COS1-del15Fw	GCTGGTCGCACCTTAAATGTAAACCAACGTTACCAGCTGAAGCTTCGTACGC
COS1-del15Rv	AGTAGTCTCGTTTTGGTTATTCGCAGCTGACAAACTACTCGATGAATTCGAGCTCGTT
COS10-del16Fw	TTACGTACCTCATAACATTAATGTGAAATACACTATATTGCCAGCTGAAGCTTCGTACGC

COS10-del16Rv	CCTTGTAGCTTCTAAGTCAAAATAAATGTTTCTATCTTTATCGATGAATTCGAGCTCGTT
COS2-Diag_Fw2	CACAAACCATCGTTCAGAAT
COS2-Diag_Rv2	TATTGTTGCCCTTTTTCTA
COS7-Diag_Fw4	CTACTAGATGTTTACACA
COS7-Diag_Rv4	ACAATACTGGAAAATCACTC
COS4-Diag_Fw7	CGAATAGTAGAGTTTCTGTG
COS4-Diag_Rv7	CGTTCAGAATATGCATGGAA
COS6-Diag_Fw8	CGGTAAAGGCTTTCATAAGC
COS6-Diag_Rv8	CCATGTGATAATGAAATTCT
COS5-Diag_Fw11	GGCAAACGATCCTTCACCTA
COS5-Diag_Rv11	GTGACAGCTACGTCTATTTT
COS9-Diag_Fw12	CCGACTTGCAATACTAAACG
COS9-Diag_Rv12	CTATTTATTGCAATTTATTC
COS3-Diag_Fw14	CTGTACTCTATAGTCATA
COS3-Diag_Rv14	CCTTCGGATTAAGTGAGGCG
COS1-Diag_Fw15	CGTAGGACAGACTCTTCCTG
COS1-Diag_Rv15	CCTTGCTTGATAAAAACACC
COS10-Diag_Fw16	CGAAACTGCTCGAAGCTGTT
COS10-Diag_Rv16	CCTTGTAGCTTCTAAGTCAA

Primers for *SIR3* deletion

Primer	Sequence
SIR3_F1	GTTTAAGAAAGTTGTTTTGTTCTAACAATTGGATTAGCTAAAATGccagctgaagcttcgtacgc
SIR3_R2	CATAGGCATATCTATGGCGGAAGTGAAAATGAATGTTGGTGGTCAtcgatgaattcgagctcgtt
SIR3_A	GCAATGACTGATACACAAAGAAATG
SIR3_B	TGTTCGTAGTACAGTTTTGGTTTGA

Primers for NuSA

Primer	Sequence	Middle of amplicon (Promoter coordinate)	5'/3' end	Size (bp)
A0	TGATTTCTTCTCGAGTCATATATAC	-649	-700	103
A0	ATATCTCGACCTGTATGAATTGT		-597	
A1	GTTGTTTTCTTTAGAATGTCTTATC	-603	-652	99
A1	TTTGTATACTTTTTTTCTCATCCTAG		-553	
A2	ACAATTCATACAGGTCGAGATAT	-574	-620	92
A2	TTTGGTGTGTTTATTTGCCTGC		-528	
A3	CTAGGATGAGAAAAAAAGTATACAAA	-533	-578	91
A3	CCAATAACTAACCAAAACTTTAAAAG		-487	
A4	GCAGGCAAATAAACACACCAAA	-504	-550	92
A4	TCTCTGTTGTCGTTAAATGCATG		-458	
A5	CTTTTAAAGTTTTGGTTAGTTATTGG	-462	-512	101
A5	ACATAGTACTGCTTTTTACATTGTA		-411	
A6	CATGCATTTAACGACAACAGAGA	-432	-480	96
A6	TCTGCAATTGAGGCAGGATTTAT		-384	
A7	ACAATGTAAAAAGCAGTACTATGT	-393	-434	83
A7	TATCTTATTTAGATTACGCATACCTTT		-351	
A8	ATAAATCCTGCCTCAATTGCAGA	-359	-407	96

A8	TGATCTGAGAGAAGTTAGTGATAT		-311	
A9	AAAGGTATGCGTAATCTAAATAAGATA	-330	-378	97
A9	GTCCAGAAATCGCTCCTTTAAAT		-281	
A10	ATATCACTAACTTCTCTCAGATCA	-290	-334	88
A10	TAGGATGTAAGTAGTCTATTTCTTTT		-246	
A11	ATTTAAAGGAGCGATTTCTGGACT	-263	-304	83
A11	AAAAGCGTTTTATTCTCTGACCTT		-221	
A12	AAAAGAAATAGACTACTTACATCCTAT	-227	-272	90
A12	TCTCGTATTTTTCTTCCATATGGT		-182	
A13	AAGGTCAGAGAATAAAACGCTTTT	-194	-244	101
A13	TCTGGGGATTCCTTTAGCAC		-143	
A14	ACCATATGGAAGAAAAATACGAGA	-162	-205	87
A14	GATATTACTTATCTCAGCTGAGG		-118	
A15	AGTGCTAAAGGAATCCCCAG	-121	-163	84
A15	ATTTTCCCTCTGCTCATTGTTC		-79	
A16	CCTCAGCTGAGATAAGTAATATC	-96	-140	89
A16	ACAAGTAGGAATGCGAAAGGAT		-51	
A17	GAACAATGAGCAGAGGGAAAAT	-74	-100	116
A17	TTTTGTTGCTACTATTTCACATTTGA		-16	
A18	ATCCTTTCGCATTCCTACTTGT	-27	-72	90
A18	AGTAGGTCCAAATATCATTGAAGAT		18	
A19	TCAAATGTGAAATAGTAGCAACAAAA	4	-41	90
A19	TTGACTTGGCCGAGCACTTA		49	
A20	ATCTTCAATGATATTTGGACCTACT	35	-7	84
A20	GCTTGGCGGTGTCTTTAATG		77	
A21	TAAGTGCTCGGCCAAGTCAA	71	30	81
A21	TAACATAACTTTGATCCTTACTCGT		111	
A22	CATTAAAGACACCGCCAAGC	94	57	73
A22	GAGGAATGATCTTGGAAATCGAT		130	
A23	ACGAGTAAGGATCAAAGTTATGTTA	125	87	75
A23	AAAAACTCACAGTTATCTCTAACAT		162	
A24	ATGTTAGAGATAACTGTGAGTTTTT	153	106	94
A24	GCAAAGAAAGGACATATTGAATCA		200	
A25	ATCGATTTCCAAGATCATTCCTC	190	140	99
A25	TGCCTGTAAATAGAAAGATAATATG		239	
A26	TGATTCAATATGTCCTTTCTTTGC	222	176	91
A26	TTTTGTAAGAAATGAAATAGAAAGGA		267	
VCX1Fw	TGCGTGTGCATCCCTACTGA	N/A (control)	N/A	68
VCX1Rv	AAGTGGTCTTCCTTGCCATGA			

Primers for RT-qPCR

Primer	Sequence
qSC_ACT1_d_F1	CTCCTACGTTGGTGATGAAGCT
qSC_ACT1_d_R2	CATATCGTCCCAGTTGGTGACA
qSC_COS12_F2	CGCCAATACTGTTCTCAGATGGT
qSC_COS12_R3	CTGACAAAGACGCTTGCGAAGA
qSC_YFR057W_F1	AGCTTCCAATATCACGAGTAAGGATC
qSC_YFR057W_R2	TTGCCACGCAAAGAAAGGACAT
